# Generalizability of Randomized Controlled Trials to Routine Clinical Care in Ulcerative Colitis

**DOI:** 10.1093/ibd/izaf012

**Published:** 2025-01-30

**Authors:** Tarun Chhibba, Alexandra Frolkis, Levi R Stein, Sangmin Lee, Kaela Schill, Elena Mitevska, Allap K Judge, Marie-Louise Martin, Meaghan Martin, Kerri L Novak, Cathy Lu, Richard J M Ingram, Melissa M Chan, Tushar Shukla, Cynthia H Seow, Gilaad G Kaplan, Ashwin N Ananthakrishnan, Remo Panaccione, Christopher Ma

**Affiliations:** Division of Gastroenterology, Department of Medicine, University of Toronto, 6 Queen’s Park Crescent West, Third Floor, Toronto, ON M5S 3H2, Canada; Division of Gastroenterology and Hepatology, Department of Medicine, University of Calgary, 6th Floor Cal Wenzel Precision Health Building, 3280 Hospital Drive NW, Calgary, AB T2N 4Z6, Canada; Inflammatory Bowel Disease Unit, Division of Gastroenterology and Hepatology, Department of Medicine, University of Calgary, 5th Floor Cal Wenzel Precision Health Building, 3280 Hospital Drive NW, Calgary, AB T2N 4Z6, Canada; Department of Medicine, Western University, 1151 Richmond Street, London, ON N6A 5C1, Canada; Cumming School of Medicine, University of Calgary, 3330 Hospital Drive NW, Calgary, AB T2N 2T8, Canada; Department of Pediatrics, University of Alberta, Edmonton Clinic Health Academy, 11405-87 Avenue, Edmonton, AB T6G 1C9, Canada; Cumming School of Medicine, University of Calgary, 3330 Hospital Drive NW, Calgary, AB T2N 2T8, Canada; Inflammatory Bowel Disease Unit, Division of Gastroenterology and Hepatology, Department of Medicine, University of Calgary, 5th Floor Cal Wenzel Precision Health Building, 3280 Hospital Drive NW, Calgary, AB T2N 4Z6, Canada; Inflammatory Bowel Disease Unit, Division of Gastroenterology and Hepatology, Department of Medicine, University of Calgary, 5th Floor Cal Wenzel Precision Health Building, 3280 Hospital Drive NW, Calgary, AB T2N 4Z6, Canada; Inflammatory Bowel Disease Unit, Division of Gastroenterology and Hepatology, Department of Medicine, University of Calgary, 5th Floor Cal Wenzel Precision Health Building, 3280 Hospital Drive NW, Calgary, AB T2N 4Z6, Canada; Inflammatory Bowel Disease Unit, Division of Gastroenterology and Hepatology, Department of Medicine, University of Calgary, 5th Floor Cal Wenzel Precision Health Building, 3280 Hospital Drive NW, Calgary, AB T2N 4Z6, Canada; Inflammatory Bowel Disease Unit, Division of Gastroenterology and Hepatology, Department of Medicine, University of Calgary, 5th Floor Cal Wenzel Precision Health Building, 3280 Hospital Drive NW, Calgary, AB T2N 4Z6, Canada; Inflammatory Bowel Disease Unit, Division of Gastroenterology and Hepatology, Department of Medicine, University of Calgary, 5th Floor Cal Wenzel Precision Health Building, 3280 Hospital Drive NW, Calgary, AB T2N 4Z6, Canada; Inflammatory Bowel Disease Unit, Division of Gastroenterology and Hepatology, Department of Medicine, University of Calgary, 5th Floor Cal Wenzel Precision Health Building, 3280 Hospital Drive NW, Calgary, AB T2N 4Z6, Canada; Inflammatory Bowel Disease Unit, Division of Gastroenterology and Hepatology, Department of Medicine, University of Calgary, 5th Floor Cal Wenzel Precision Health Building, 3280 Hospital Drive NW, Calgary, AB T2N 4Z6, Canada; Department of Community Health Sciences, University of Calgary, 2500 University Drive NW, Calgary, AB T2N 1N4, Canada; Inflammatory Bowel Disease Unit, Division of Gastroenterology and Hepatology, Department of Medicine, University of Calgary, 5th Floor Cal Wenzel Precision Health Building, 3280 Hospital Drive NW, Calgary, AB T2N 4Z6, Canada; Department of Community Health Sciences, University of Calgary, 2500 University Drive NW, Calgary, AB T2N 1N4, Canada; Division of Gastroenterology, Massachusetts General Hospital and Harvard Medical School, 275 Cambridge Street, Boston, MA 02114, USA; Inflammatory Bowel Disease Unit, Division of Gastroenterology and Hepatology, Department of Medicine, University of Calgary, 5th Floor Cal Wenzel Precision Health Building, 3280 Hospital Drive NW, Calgary, AB T2N 4Z6, Canada; Inflammatory Bowel Disease Unit, Division of Gastroenterology and Hepatology, Department of Medicine, University of Calgary, 5th Floor Cal Wenzel Precision Health Building, 3280 Hospital Drive NW, Calgary, AB T2N 4Z6, Canada; Department of Community Health Sciences, University of Calgary, 2500 University Drive NW, Calgary, AB T2N 1N4, Canada

**Keywords:** trials, inclusion, biologic, molecule, ulcerative colitis

## Abstract

**Background:**

Historically, randomized controlled trials (RCTs) have been criticized for being poorly generalizable to patients with ulcerative colitis (UC) evaluated in routine care. We aimed to evaluate the proportion of patients with UC starting an advanced therapy who would be eligible to participate in phase 3 registrational UC RCTs.

**Methods:**

We conducted a retrospective cohort analysis of UC patients starting vedolizumab, ustekinumab, or tofacitinib at 2 IBD clinics at the University of Calgary. Patient charts, endoscopy reports, and laboratory results were reviewed, and compared against the inclusion and exclusion criteria from 5 RCTs (GEMINI-I, UNIFI, OCTAVE, ELEVATE, and LUCENT). The proportion of patients who would have been deemed eligible versus ineligible for trial participation at the time of starting a new advanced therapy was determined.

**Results:**

A total of 125 patients with UC were included: 78 (62.4%) would have been eligible for at least one of the considered RCTs. Trial-eligible patients were younger, less likely to be exposed to prior immunosuppressants, and had higher C-reactive protein and fecal calprotectin. The most common reason for trial ineligibility was having inadequate disease activity at baseline (Mayo endoscopy subscore <2 or absence of rectal bleeding). A significantly greater proportion of patients would have been eligible for LUCENT (45.6%) compared to GEMINI-I (24.8%), OCTAVE (35.2%), or ELEVATE (35.2%) (*P* < .01 for all comparisons).

**Conclusions:**

Half of patients with UC starting advanced therapy in routine care may be eligible for participation in phase 3 RCTs. Disease activity is the primary reason for trial exclusion.

**Key Messages**

**What Is Already Known?**

Randomized controlled trials (RCTs) assess the safety and efficacy of novel therapeutic options in the treatment of ulcerative colitis (UC) but may exclude certain populations.

**What Is New Here?**

Half of patients started on advanced therapies for UC in clinical practice would be eligible for participation in RCTs based on trial inclusion and exclusion criteria, with insufficient disease activity being the most common reason for ineligibility.

**How Can This Study Help Patient Care?**

As more advanced therapies for UC become available, it is crucial that results are generalizable. Identifying the reasons for ineligibility is an important step toward optimizing the design of RCTs and guiding clinical decision making.

## Introduction

Ulcerative colitis (UC) is an immune-mediated inflammatory disease of the rectum and colon characterized by bloody diarrhea, urgency, and abdominal pain.^1^ Over the past several decades, the introduction of advanced targeted immune modulators (TIMs), including biologic agents and orally administered small molecules, have improved the management of moderately-to-severely active UC.^2^ Optimizing medical therapy to control luminal inflammation and achieve endoscopic remission is currently recommended by clinical practice guidelines and is associated with improved quality of life and lower risks of long-term disease-related complications, including hospitalization, colectomy, incontinence, and potentially, colorectal dysplasia.^3–6^ Currently, multiple classes of biologics targeting tumor necrosis factor (TNF) alpha, α_4_β_7_ integrin, interleukin (IL)-12/23p40, and IL23p19, as well as 2 classes of advanced oral small molecules targeting Janus kinase (JAK) and sphingosine-1-phosphate (S1P) receptors are currently licensed for the treatment of moderately-to-severely active UC.^7^

The approval of novel therapies depends on data from registrational phase 3 randomized controlled trials (RCTs). Notably, these are designed as explanatory RCTs, which feature strict inclusion and exclusion criteria, rigid treatment protocols, and measurement of pre-specified outcome measures that can support subsequent regulatory approval.^8^ While this study design has been optimized for drug development, these trials are often criticized for being poorly generalizable to routine clinical care and inadequately reflect the diversity of patients managed in day-to-day practice. For example, Ha et al. previously showed that only 1 in 3 patients with inflammatory bowel disease (IBD) in routine clinical practice would have qualified for the pivotal trials evaluating the efficacy and safety of TNF antagonists,^9^ and similar findings have been shown in RCTs of other chronic inflammatory diseases.^10,11^

Recognizing these limitations, there has been an increased focus on designing more patient-centric clinical trials in IBD: many studies have broadened inclusion criteria and now allow the enrollment of participants with prior advanced treatment exposures, trial schedules have been simplified and are increasingly structured to facilitate remote visits, and primary trial endpoints highlight the use of both objective and patient-reported outcome measures.^12,13^ In this study, we evaluated the eligibility of UC patients in routine care for inclusion in more recent registrational UC RCTs based on their demographics and disease characteristics to assess how inclusion and exclusion criteria may impact the applicability and generalizability of RCT results to clinical practice.

## Methods

### Study Design

We conducted a retrospective cohort study of patients with a confirmed diagnosis of UC starting vedolizumab, ustekinumab, or tofacitinib as part of their routine clinical care, at 2 ambulatory IBD clinics at the University of Calgary, from January 2018 to December 2021. Both clinics are part of the Calgary Zone of Alberta Health Services, a publicly funded, single-payer healthcare system. The health system uses a unified electronic medical record, capturing all medical encounters, clinical notes, laboratory information, pharmaceutical prescriptions, and endoscopy and histopathology results. We evaluated whether these patients starting an advanced therapy as part of their clinical care would have been eligible for 5 different phase 3 RCTs, and selected trials based on capturing advanced therapies with different mechanisms of action: GEMINI-I (Vedolizumab as induction and maintenance therapy for UC),^14^ UNIFI (Ustekinumab as induction and maintenance therapy for UC),^15^ OCTAVE (Tofacitinib as induction and maintenance therapy for UC),^16^ ELEVATE (Etrasimod as induction and maintenance therapy for UC),^17^ and LUCENT (Mirikizumab as Induction and Maintenance Therapy for UC).^18^ We specifically chose phase 3 trials that are required for regulatory approval, and therefore, are generally the most directly relevant to clinical care. We chose one trial from each class of available TIM that is currently part of the therapeutic armamentarium, because patients considering participation in an RCT are often faced with the decision of whether they should be treated with an investigational product or receive a currently marketed treatment. The inclusion and exclusion criteria for each study were obtained through the study protocols, publications, investigator brochures, and registered data on *Clinicaltrials.gov* (Supplementary Appendix 1).

### Data Collection

Patients initiated on ustekinumab, vedolizumab, or tofacitinib for management of UC were eligible to be included in this study. Patients were excluded if they had a diagnosis of Crohn’s disease, indeterminate colitis, or started their advanced TIM for a non-IBD indication (eg, patients starting tofacitinib for management of rheumatoid arthritis in the context of having UC were excluded). We excluded patients who had insufficient records at the time of treatment initiation or were started on TIM outside of the University of Calgary IBD Clinic (eg, patients starting treatment in a private medical clinic). All data were collected using a standardized case report form.

Demographic data were collected for all patients including sex, year of birth, age at diagnosis, body mass index, UC disease extent, UC treatment history, medical and surgical history (including all relevant comorbidities that would be exclusionary for trial participation), social history including alcohol, smoking and substance use, and laboratory data. Clinical disease activity assessed within 12 weeks prior to initiating advanced TIM was used to determine eligibility, with the closest clinical visit and endoscopic assessment prior to the exact date of initiation being used to score disease activity. If no clinical assessments were included within 3 months of TIM initiation, patients were excluded. Disease activity was collected using the modified Mayo Score (mMS), comprising the rectal bleeding, stool frequency, and endoscopic appearance subscores. We did not collect the physician global assessment (PGA), given the subjectivity and inter-observer variability in this parameter, and the inability to accurately capture this retrospectively.

### Outcomes and Analysis

The primary outcome of interest was the proportion of patients starting advanced TIM therapy in routine care who would have met eligibility for at least one phase 3 RCT. To be considered eligible, the patient needed to meet all inclusion and exclusion criteria. Three of the considered trials (GEMINI-I, UNIFI, OCTAVE) used the full Mayo Clinic score (MCS, including the PGA) for eligibility, requiring a baseline MCS ≥6 for enrollment. For this study, we considered patients with an mMS ≥5 to be eligible as the PGA was not collected. Reasons for ineligibility were broadly classified as those relating to: (1) disease-related parameters (exclusion based on inadequate or too severe clinical or endoscopic disease activity); (2) patient-related parameters (exclusion based on medical or surgical comorbidities); and (3) protocol-related parameters (exclusion based on prior impermissible treatment exposures or other protocol violations). As a secondary analysis, we also evaluated the proportion of participants who would be eligible based on disease activity alone, using the 2022 US Food and Drug Administration draft guidance.^19^

Demographic characteristics were summarized using descriptive statistics. Comparisons of proportions were made using the Pearson *χ*^2^ test and comparisons of continuous covariables were assessed using the two-sample *t*-test. All analyses were conducted in STATA 17.0 (StataCorp, College Station, TX).

## Results

### Patient Population

Overall, we included 125 patients who were initiated on vedolizumab (64/125, 51.2%), ustekinumab (33/125, 26.4%), or tofacitinib (28/125, 22.4%) for management of UC (Table 1). Mean age was 41.8 years (standard deviation SD 17.2 years) and mean disease duration was 10.4 years (SD 9.4 years). Approximately two-thirds of patients (63.2%) were exposed to a previous biologic or small molecule therapy, with most having previously been treated with TNF antagonist therapy prior to switching to an alternative TIM (72/125, 57.6%). A total of 30.4% (38/125) patients received corticosteroids at the time of TIM initiation. Almost all patients (115/125, 92.0%), of patients starting a TIM, had evidence of an MES ≥ 1, fecal calprotectin ≥ 250 µg/g, or CRP > 5 mg/L.

**Table 1.** Baseline demographics and disease characteristics of ulcerative colitis patients starting advanced therapy.

Characteristic	*n* = 125
Treatment, *n* (%)	
Vedolizumab	64 (51.2)
Ustekinumab	33 (26.4)
Tofacitinib	28 (22.4)
Male sex, *n* (%)	65 (52.0)
Mean age, years (SD)	41.8 (17.2)
Mean disease duration, years (SD)	10.4 (9.4)
Disease extent, *n* (%)	
Pancolitis	62 (49.6)
Left-sided colitis	50 (40.0)
Proctitis	13 (10.4)
Modified Mayo Score (mMS)	
Mean (SD)	5.3 (2.2)
mMS ≥ 4 (%)	97 (77.6)
mMS ≥ 5 (%)	79 (63.2)
Endoscopic appearance, *n* (%)	
Mildly active disease (MES = 1)	21 (16.8)
Moderately active disease (MES = 2)	38 (30.4)
Severely active disease (MES = 3)	51 (40.8)
Smoking status, *n* (%)	
nonsmoker	106 (84.8)
Current smoker	18 (14.4)
Prior treatment exposure, *n* (%)	
Prior 5-aminosalicylate therapy	106 (84.8)
Prior immunosuppressant use	57 (45.6)
Prior advanced therapy	79 (63.2)
Prior advanced treatment exposure, *n* (%)	
1 prior advanced TIM	32 (25.6)
2 prior advanced TIM	31 (24.8)
≥ 3 prior advanced TIM	16 (12.8)
Prior classes of advanced treatment, *n* (%)	
Prior TNF antagonist	72 (57.6)
Prior vedolizumab	37 (29.6)
Prior ustekinumab	6 (4.8)
Prior tofacitinib	7 (5.8)
Baseline biochemistry	
Mean CRP (mg/L)	10.0 (18.0)
CRP > 5 mg/L (%)	33 (38.8)
Mean fecal calprotectin (µg/g)	886.7 (871.9)
Fecal calprotectin > 250 µg/g (%)	13 (59.0)
Mean hemoglobin (g/L)	135.4 (18.1)
Mean albumin (g/L)	35.6 (5.0)

Abbreviations: CRP, C-reactive protein; MES, Mayo endoscopy subscore; SD, standard deviation; TIM, targeted immune modulator; TNF, tumor necrosis factor.

### Overall Trial Eligibility

Of the 125 patients, 78 (62.4%) would have been eligible for at least one of the considered RCTs. Table 2 summarizes the characteristics of patients who were ineligible for all trials compared to patients who met eligibility for at least one RCT. Patients who were trial eligible tended to be younger (mean Δ5.42 years, *P* = .04), and were less likely to be exposed to prior immunosuppressants (35.9% vs 61.7%, *P* = .01). Patients who were trial eligible also had higher mean CRP (Δ8.6 mg/L, *P* = .04) and fecal calprotectin (Δ430.5 µg/g, *P* = .03), and had lower mean hemoglobin (Δ −9.8 g/L, *P* = .02) and albumin (Δ −2.6 g/L, *P* = .03). There was also a significant difference in baseline endoscopic disease activity: all 21 patients with mild endoscopic disease activity (MES = 1) were trial-ineligible, whereas 60.3% (47/78) of trial-eligible patients had severe MES = 3 (*P* < .001). The mean modified Mayo score (mMS) was 3.4 (SD 1.8) for trial-ineligible patients compared to 6.5 (SD 1.5) for trial-eligible patients (*P* < .001).

**Table 2.** Characteristics of participants who were ineligible for all clinical trials.

Characteristics	Ineligible for all trials (*n* = 47)	Eligible for at least one trial (*n* = 78)	*P* value
Male sex, *n* (%)	22 (46.8)	43 (55.1)	.37
Mean age, years (SD)	45.2 (18.4)	39.8 (16.1)	.04
Mean disease duration, years (SD)	9.5 (7.8)	10.9 (10.3)	.79
Disease extent, *n* (%)			
Proctitis only	5 (10.6)	8 (10.4)	.08
Left-sided colitis	13 (27.7)	37 (47.4)	
Pancolitis	29 (61.7)	33 (42.3)	
Mean Modified Mayo Score (SD)	3.4 (1.8)	6.5 (1.5)	<.001
Endoscopic appearance, *n* (%)			
Mildly active disease (MES = 1)	21 (44.7)	0 (0)	<.001
Moderately active disease (MES = 2)	7 (14.9)	31 (39.7)	
Severely active disease (MES = 3)	4 (8.5)	47 (60.3)	
Smoking status, *n* (%)			
nonsmoker	42 (89.4)	65 (83.3)	.47
Current smoker	5 (10.6)	13 (16.7)	
Prior treatment exposure, *n* (%)			
Prior 5-aminosalicylate therapy	39 (83.0)	67 (85.9)	.66
Prior immunosuppressant use	29 (61.7)	28 (35.9)	.01
Prior corticosteroids	36 (76.6)	65 (83.3)	.35
Prior advanced therapy	26 (55.3)	53 (67.9)	.16
Prior advanced treatment exposure, *n* (%)			
1 prior advanced TIM	12 (25.5)	20 (25.6)	.40
2 prior advanced TIM	9 (19.2)	22 (28.2)	
≥3 prior advanced TIM	5 (10.6)	11 (14.1)	
Baseline biochemistry, mean (SD)			
C-reactive protein (mg/L)	4.4 (6.2)	13.0 (21.3)	.04
Fecal calprotectin (µg/g)	599.7 (746.4)	1030.3 (919.8)	.03
Hemoglobin (g/L)	141.7 (17.4)	131.9 (17.7)	.02
Albumin (g/L)	37.3 (3.9)	34.7 (5.3)	.03

Abbreviations: MES, Mayo endoscopy subscore; SD, standard deviation; TIM, targeted immune modulator.

When considering the 2022 US FDA draft guidance, 57.6% (72/125) of patients would have been considered eligible for inclusion in trials evaluating novel therapies for moderately-to-severely active UC based on having a mMS 5-9 and MES ≥ 2. Most ineligible patients were excluded based on having inadequate endoscopic disease activity (67.9%, 36/53) whereas 17 patients (32.1%) had MES = 2/3 but inadequate stool frequency and rectal bleeding subscores to qualify for enrollment. As a sensitivity analysis, we considered several other potential trial eligibility scenarios. When using mMS 4-9 as the inclusion criteria, 68.8% (86/125) of patients would have been considered eligible and only 3 patients (7.7%) with MES = 2 would have been excluded based on inadequate patient-reported outcome subscores. Allowing patients with isolated proctitis but meeting other eligibility criteria increases the proportion of eligible patients from 50.4% to 57.6%. Allowing patients to enroll on the basis of only at least moderate endoscopic disease activity (MES ≥ 2) without specific PRO requirements would allow 71.2% (89/125) to participate.

### Eligibility for Specific Trials

**Figure 1.**
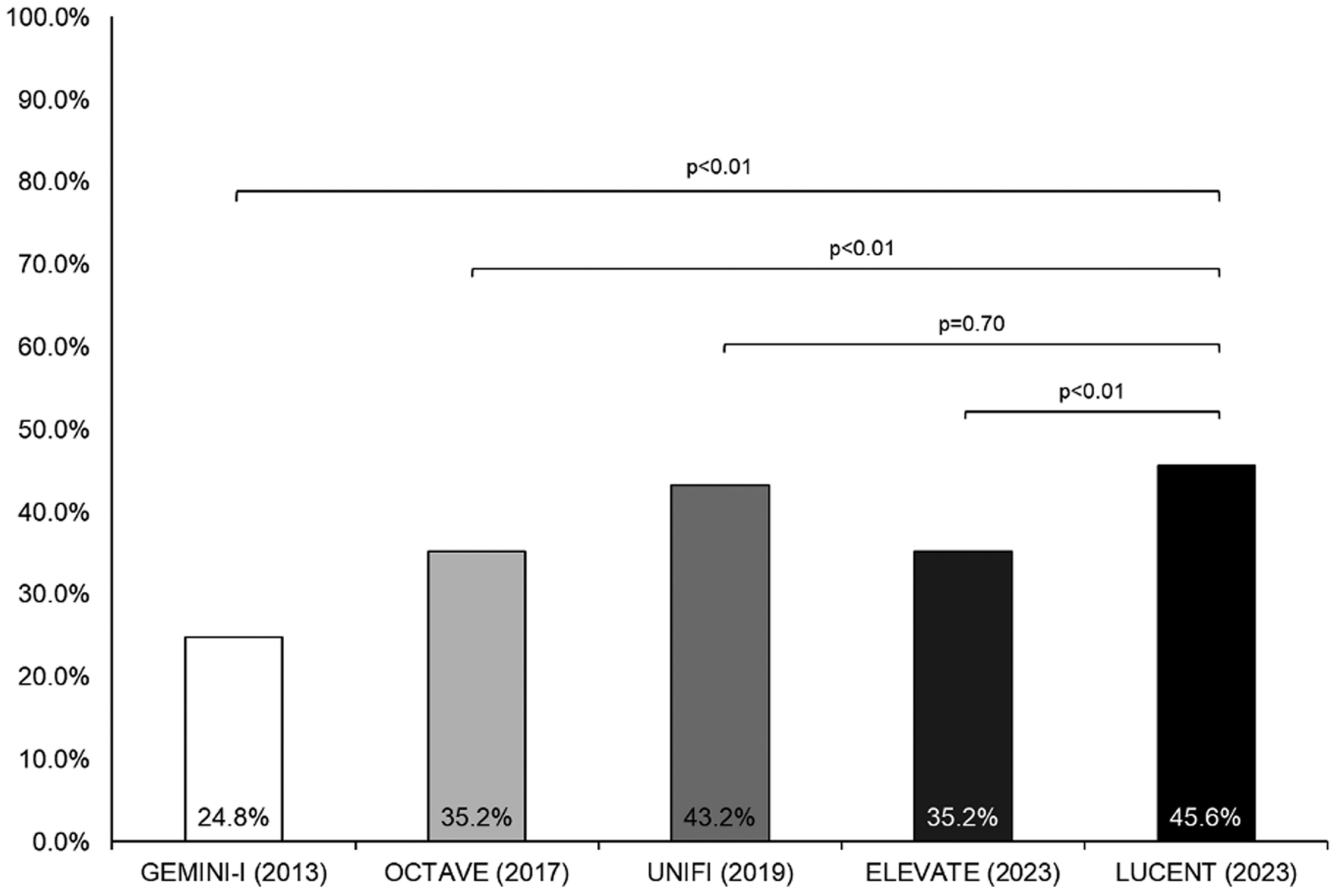
Proportion of patients starting advanced therapy in routine care that would have been eligible for phase 3 registrational trials in ulcerative colitis. GEMINI-I: Vedolizumab as Induction and Maintenance Therapy for Ulcerative Colitis; OCTAVE: Tofacitinib as Induction and Maintenance Therapy for Ulcerative Colitis; UNIFI: Ustekinumab as Induction and Maintenance Therapy for Ulcerative Colitis; ELEVATE: Etrasimod as induction and maintenance therapy for ulcerative colitis (ELEVATE): 2 randomized, double-blind, placebo-controlled, and phase 3 studies; LUCENT: Mirikizumab as Induction and Maintenance Therapy for Ulcerative Colitis.

Overall, 24.8% (31/125) of patients would have been eligible for inclusion in GEMINI-I, 35.2% (44/125) patients were eligible for OCTAVE, and 43.2% (54/125) patients were eligible for UNIFI (Figure 1). The most common reasons for exclusion are summarized in Tables 2 and 3. The most common reasons for exclusion were disease-related factors, which were present in approximately half of patients, including newly diagnosed UC (<6 months of confirmed disease) or inadequate endoscopic disease activity. A total of 11 patients were starting an advanced treatment as their first-line therapy and would have been excluded and 13 patients had isolated proctitis, which was an exclusion criterion. Patient-related factors, including adolescent or elderly (>80 years) age or exclusionary comorbidities were prevalent in approximately one-quarter of patients. Prior vedolizumab exposure excluded 29.5% (37/125) patients from participation in GEMINI-I. Of the 2 most recently published trials, 35.2% (44/125) patients were eligible for ELEVATE and 45.6% (57/125) were eligible for LUCENT. The most common reasons for exclusion in ELEVATE were having an mMS < 4 (*n* = 28) or absence of rectal bleeding (*n* = 51). A significantly greater proportion of patients would have been eligible for LUCENT as compared to GEMINI-I, OCTAVE, and ELEVATE (*P* < .01 for all comparisons).

**Table 3.** Reasons for trial ineligibility.

Trial	GEMINI-I	OCTAVE	UNIFI	ELEVATE	LUCENT
Patient-related exclusions	Age < 18 or > 80 (*n* = 3) Previous extensive colonic resection (*n* = 1) Stoma (*n* = 2) Imminent surgery required (*n* = 2) History of *C. difficile* infection (*n* = 10) HBV (*n* = 1), tuberculosis (*n* = 3) Pregnancy (*n* = 1) Malignancy (*n* = 7) Neurological diagnosis (*n* = 2) History of psychiatric disease (*n* = 3)	Age < 18 (*n* = 1) Microscopic colitis (*n* = 2) Fulminant colitis (*n* = 1)	Age < 18 (*n* = 1) Previous resection or stoma (*n* = 3) Imminent surgery required (*n* = 2) History of colonic dysplasia (*n* = 4) Microscopic colitis (*n* = 2) Stool infection (*n* = 10) HBV (*n* = 1) Malignancy history (*n* = 7) Alcohol abuse (*n* = 1) Pregnancy (*n* = 1)	Age > 80 (*n* = 3) Toxic megacolon/fulminant colitis (*n* = 1) Colonic resection/ostomy (*n* = 3) Imminent surgery required (*n* = 2) Dysplasia (*n* = 4) Microscopic colitis (*n* = 2) TB (*n* = 3) Malignancy (*n* = 7) HBV (*n* = 1) Herpes zoster (*n* = 3) Alcohol abuse (*n* = 1) Neurological disease (*n* = 2) Pregnancy (*n* = 1) History of MI/CVA (*n* = 2) Cardiac disease (*n* = 4) Pulmonary disease (*n* = 1)	Age < 18 (*n* = 1) or > 80 (*n* = 3) Pregnancy (*n* = 1) Colonic resection (*n* = 1) Dysplasia (*n* = 4) TB (*n* = 3), HBV (*n* = 1), zoster (*n* = 3) Malignancy (*n* = 7) Neurological disease (*n* = 2)
Disease-related exclusions	UC < 6 months (*n* = 7) MCS < 6 (*n* = 34) MES < 2 (*n* = 36) Proctitis (*n* = 13)	UC < 4 months (*n* = 6) MCS < 6 (*n* = 34) MES < 2 (*n* = 36) Proctitis (*n* = 13) Absence of rectal bleeding (*n* = 51) Absence of failure of other therapies (*n* = 11)	UC < 3 months (*n* = 5) MCS < 6 (*n* = 34) MES < 2 (*n* = 36) Proctitis (*n* = 13) No failure of prior immunomodulator/steroids/vedo/TNF (*n* = 11)	UC < 3 months (*n* = 5) mMS < 4 (*n* = 28) MES < 2 (*n* = 36) RB < 1 (*n* = 51) Advanced treatment failure (*n* = 16)	UC < 3 months (*n* = 5) mMS < 4 (*n* = 28) MES < 2 (*n* = 36) Proctitis (*n* = 13) No prior failure of therapy (*n* = 11)
Protocol-related exclusions	Previous vedolizumab exposure (*n* = 37) Other protocol violations (*n* = 2)	Previous tofacitinib exposure (*n* = 7) Other protocol violations (*n* = 2)	Previous ustekinumab exposure (*n* = 6) Other protocol violations (*n* = 3)	Other protocol violations (*n* = 6)	Prior ustekinumab exposure (*n* = 6) Other protocol violations (*n* = 3)

## Discussion

The generalizability of RCTs impacts the interpretation and application of trial results to clinical practice. In this retrospective cohort study of 125 patients with UC starting on a new advanced therapy, we demonstrate that over half would be potentially eligible for at least one of the major registrational trials conducted over the past decade. The most common reason for trial ineligibility is inadequate disease activity meeting the thresholds for enrollment in an RCT, despite having an indication to switch or start advanced treatment in clinical care. These findings are important for considering the overall generalizability of pivotal trial findings, as well as evaluating the inclusion and exclusion criteria for future studies.

Historically, registrational trials have captured the minority of patients with IBD seen in clinical care: in a study from 2011, Ha et al. examined the eligibility of 81 UC patients who required escalation or adjustment of their medical treatment, demonstrating that only 1 in 4 patients would have been eligible to participate in the ACT 1 or ACT 2 trials of infliximab for UC.^9^ Common reasons for exclusion were use of topical rescue therapy and requirement for colectomy (due to age, comorbidities, or dysplasia), and 1 in 3 patients were excluded due to TNF antagonist exposure. Notably, there have been substantive changes in clinical care paradigms since this study was originally published, including the introduction of multiple classes of advanced TIMs, improvements in endoscopic dysplasia management,^20^ and a greater focus on tight disease control through noninvasive biomarker monitoring and treating to endoscopic remission as a therapeutic target.^21^

How have these advancements changed the profile of patients participating in contemporary RCTs? In our study, we show that the primary reason for patient ineligibility in current UC trials is inadequate disease activity. This corroborates similar findings from an analysis of 107 patients with IBD evaluated for clinical trial participation at IOIBD sites, where the most common reason for screen failure was insufficient disease activity.^22^ Hence, an interesting paradox must be solved for future trials: having a higher threshold at baseline hampers recruitment and reduces trial generalizability, yet results in the randomization of patients with more substantial disease activity, which is critical for improving sensitivity for detecting treatment efficacy signals. This was demonstrated by Feagan et al. in a seminal study of mesalamine, where excluding patients without at least moderate baseline endoscopic inflammation, confirmed by an objective, blinded central reader, increased the Week 10 remission rate from 13.8% to 29.0%.^23^

Our study highlights a potential disconnect between the current dogma in IBD management of “treating-to-target” and the minimum disease activity required to enroll in clinical trials. Most patients in our study were started on an advanced TIM due to the presence of active disease, defined by a MES ≥ 1, CRP ≥ 5 mg/L, or fecal calprotectin ≥ 250 µg/g, consistent with STRIDE-II recommendations that normalization of biomarkers and achievement of endoscopic remission are appropriate intermediate and long-term therapeutic targets, respectively.^6^ In contrast, most clinical trials require demonstration of “moderately-to-severely” active disease and therefore, there are patients who may have not yet met STRIDE-II defined targets, yet have active disease not meeting the inclusion criteria for most RCTs. How should this subset of patients be managed? Treatment optimization to achieve deeper levels of remission may be appropriate in clinical care, although we note that well-controlled, prospective trials that definitively answer the questions of whether switching class or optimizing therapy to target endoscopic or histologic remission in UC are still ongoing.

The current regulatory framework uses the mMS for defining baseline eligibility.^19^ Patients in our study who were deemed ineligible for trial participation generally had lower endoscopy subscores, less biomarker evidence of inflammation, or absence of rectal bleeding. These findings highlight that while symptom-endoscopy correlation in UC may be better than in Crohn’s disease, a substantial proportion of patients will have discordant rectal bleeding and stool frequency subscores compared to their endoscopic appearance.^24–27^ In clinical care, differentiating the overlap between functional disorders of gut–brain interaction in patients with substantial symptom burden but lower objective disease markers and/or the absence of rectal bleeding can be difficult, especially as over 1/3 of UC patients can exhibit symptoms meeting criteria for irritable bowel syndrome. Accordingly, it may be reasonable to consider reducing symptom-based thresholds for trial inclusion, with some recent RCTs now enrolling patients with an mMS of ≥4 (as opposed to ≥5) provided that all participants have objectively confirmed endoscopic inflammation at baseline.^17,18^ Secondly, our findings emphasize that developing a framework that allows patients with ongoing mildly-to-moderately active UC to participate in trials is a research priority.^28^ Over half of patients in our study who were ineligible for trial participation had prior advanced therapy exposure but did not achieve clinical or endoscopic remission, hence necessitating the initiation of a new TIM in accordance with a treat-to-target strategy. These patients often experience some improvement with their index therapy and while they may not meet “conventional” inclusion criteria for moderately-to-severely active disease, they clearly have ongoing mild-to-moderate inflammation.

Although we showed that nearly two thirds of UC patients starting a TIM in routine care *could* be eligible for at least one of the considered phase 3 trials, recruitment to IBD RCTs has been increasingly difficult, averaging approximately 0.1-0.2 randomized participants per site per month.^29,30^ Vieujean et al. assessed reasons why clinical trials were not chosen as a treatment option: with more available therapies, both physicians and patients expressed preferences for using a specific approved treatment and hesitancies with participating in proscriptive trials with rigid protocols and risks of randomization to placebo.^22^ Focusing on patient-centric trial designs can help overcome some of these obstacles. For example, about 2/3 of IBD clinical trials require patients to undergo a washout period of prior advanced therapy, despite little evidence supporting the necessity for this approach.^31^ Eliminating these washout periods may improve patient willingness to screen for trials. Secondly, many trials have already prioritized broadening inclusion criteria to include patients with proctitis or with multiple prior treatment failures. This approach should be encouraged in RCTs, as patients with limited disease extent often still have significant symptoms and severely active inflammation which is refractory to topical therapies. While these groups have traditionally been considered challenging to treat, they have substantial unmet medical needs that need to be addressed, and the need for systemic therapies should not be determined by disease extent alone.^32^ Third, trial configurations that minimize risks of placebo exposure, such as within platform or Bayesian designs, balance the need for scientific rigor while balancing patient risks for participation.^12,33^ Finally, simplifying follow-up visit schedules and facilitating easier access to remote trial visits could improve access to RCTs for a broader population of UC patients.

Our study has several strengths. First, we included patients from a large and diverse practice setting, which includes both academic tertiary hospital referrals and community care, as this can influence which patients receive advanced therapy. Second, we captured comprehensive clinical, laboratory, and endoscopic data, which was available within a single health system. However, there are important limitations that should be acknowledged. First, this was a retrospective analysis whereas clinical trial assessments are done prospectively. We were, therefore, unable to capture multiple daily recall periods that are required for the calculation of the mMS in the trial setting. We started the analysis in 2018, although there may be temporal changes in who is prescribed advanced TIMs throughout the study period and when extrapolating to contemporary management. Second, all endoscopic evaluations were done by the primary gastroenterologist as part of routine care and are prone to inter-rater variability in assessment. Third, we excluded patients with inadequate information at the time of starting a TIM for defining disease activity. The proportion of these patients who would have been trial eligible is unclear. In addition, we restricted our analysis to patients starting a TIM in routine care as these would have been the most likely patients to have also been considered for a clinical trial with active disease, although we recognize there are likely many patients in routine care who may also qualify for a clinical trial but are not started on treatment for other reasons. Similarly, we evaluated eligibility only based on inclusion and exclusion criteria, but there may be patients who decline participation in trials for other reasons, such as preferences to avoid placebo or inability to adhere to invasive trial protocols. Fourth, our cohort only includes outpatients initiated on TIMs. Although hospitalized patients with UC have more severe disease activity, these patients are universally excluded from participating in ambulatory trials of moderately-to-severely active UC, given the unique phenotype and prognosis of acute severe UC.

In conclusion, over half of patients with UC starting an advanced therapy in routine care could be potentially eligible for inclusion in an RCT. The primary reasons for ineligibility relate to inadequate disease activity, highlighting that there may be circumstances in clinical practice that necessitate a change in therapy yet do not meet “traditional” trial eligibility criteria. Over time, there has been an increasing focus on maximizing generalizability of RCT results to clinical settings, and designing studies to better meet the needs of patients and prescribers may help to reduce the burden of slow recruitment in UC trials.

## Supplementary Data

Supplementary data is available at *Inflammatory Bowel Diseases* online.

izaf012_suppl_Supplementary_Appendixs
