# Supplementary material for: Generalizability of Randomized Controlled Trials to Routine Clinical Care in Ulcerative Colitis
Source: Inflamm Bowel Dis. 2025 Jan 30;31(8):2088–96. doi: 10.1093/ibd/izaf012 (PMC12342827; doi:10.1093/ibd/izaf012)
Supplement: izaf012_suppl_Supplementary_Appendixs [file izaf012_suppl_supplementary_appendixs.docx]

Supplementary Appendix 1: Inclusion and Exclusion Criteria of Randomized Controlled Trials

| Tofacitinib:   \| **Inclusion Criteria (must meet all of the following to be eligible):** \| **Exclusion Criteria (meet any of the criteria will not be eligible):** \| \| --- \| --- \| \| 1. Subject must be at least 18 years of age.  2. Males and females with a documented diagnosis (endoscopic or radiographic and histological) of UC ≥4 months prior to entry into the study. A biopsy report supporting the diagnosis must be available in the source documents.  3. Subjects with moderately to severely active UC as defined by a total Mayo score of ≥6, with a rectal bleeding score of ≥1 and an endoscopic subscore of ≥2 on the Mayo score determined within 7 days of baseline visit (Visit 2).  4. Subjects must have failed or be intolerant (discontinued the medication due to an adverse event as determined by the investigator) of at least one of the following treatments for UC:  • Oral corticosteroids.  • Azathioprine or 6-mercaptopurine (6-MP).  • Anti-TNF therapy: infliximab or adalimumab.  5. Subjects currently receiving the following treatment for UC are eligible providing they have been and are anticipated to be on stable dose for designated period of time:  • Oral 5-ASA or sulfasalazine stable dose for at least 4 weeks prior to baseline and during the study period.  • Oral corticosteroids (prednisone equivalent up to 25 mg/day; budesonide up to 9 mg/day) stable dose for at least 2 weeks prior to baseline and during the study period.  • Chronic treatment for ulcerative colitis with antibiotics (eg, metronidazole, rifaximin) stable dose for at least 2 weeks prior to baseline and during the study period.  6. No evidence of active or latent or inadequately treated infection with Mycobacterium tuberculosis (TB) as defined by all of the following:  • A negative QuantiFERON® -TB Gold (QFT-G) In-Tube test or, if unavailable or indeterminate upon retest, a Mantoux/Purified Protein Derivative (PPD) tuberculin skin test as per local medical standard of practice, with a result of < 5mm of induration, performed at or within the 3 months prior to a given Screening visit. [It is recommended that subjects with a history of Bacille Calmette Guérin (BCG) vaccination be tested with the QFT-G test].  • A chest radiograph, taken at or within the 3 months prior to a given Screening visit, without changes suggestive of active TB infection as determined by a qualified radiologist.  • No history of either untreated or inadequately treated latent or active TB infection.  • If a subject has previously received an adequate course of therapy for either latent (9 months of isoniazid in a locale where rates of primary multi-drug TB resistance are <5% or an acceptable alternative regimen) or active (acceptable multi-drug regimen) TB infection, neither a QFT-G test nor a PPD test is needed, but a chest radiograph must still be obtained if not performed within 3 months prior to a given Screening visit. Documentation of adequate treatment for TB will be obtained prior to first dose of study drug.  • A subject who is currently being treated for active TB infection is to be excluded.  • A subject who is currently being treated for latent TB infection can only be enrolled with confirmation of current incidence rates of multi-drug resistant TB infection in the locale, documentation of an adequate treatment regimen, and with prior approval by the sponsor.  7. Female subjects of childbearing potential must agree to use a highly effective method of contraception throughout the study and for at least 4 weeks after the last dose of assigned treatment. A subject is of childbearing potential if, in the opinion of the investigator, he/she is biologically capable of having children and is sexually active.  8. Women of childbearing potential must have a negative pregnancy test prior to study enrollment.  9. Subjects receiving non-prohibited concomitant medications for any reason, must be on a stable regimen, which is defined as not starting a new drug or changing dosage with 7 days or 5 half-lives (whichever is longer) prior to first study dose.  10. Subjects who are willing and able to comply with scheduled visits, treatment plan, laboratory tests, daily diary call, and other study procedures.  11. Evidence of a personally signed and dated informed consent document indicating that the subject (or a legally acceptable representative) has been informed of all pertinent aspects of the study \| 1. Presence of indeterminate colitis, microscopic colitis, ischemic colitis, infectious colitis, or clinical findings suggestive of Crohn’s disease.  2. Subjects with disease limited to distal 15 cm.  3. Subjects without previous treatment for UC (ie, treatment-naïve).  4. Subjects receiving the following therapy within the designated time period or are expected to receive any of these therapies during the study period:  • Azathioprine, 6-mercaptopurine, or methotrexate within 2 weeks prior to baseline.  • Anti-TNF therapy (eg, infliximab, adalimumab, or certolizumab) within 8 weeks prior to baseline.  • Cyclosporine, mycophenolate, or tacrolimus within 8 weeks prior to baseline. • Interferon therapy within 8 weeks prior to baseline.  • Intravenous corticosteroids within 2 weeks prior to baseline.  • Rectally administered formulation of corticosteroids or 5-ASA within 2 weeks prior to baseline.  • Anti-adhesion molecule therapy taken within 1 year (eg, natalizumab or any investigational anti-adhesion molecule therapy).  5. Subjects displaying clinical signs of fulminant colitis or toxic megacolon.  6. Subjects with evidence of colonic adenomas or dysplasia. However, subjects with prior history of adenomatous polyps will be eligible if the polyps have been completely removed and the subjects are free of polyps at baseline.  7. Subjects at risk for colorectal cancer must have a colonoscopy. 8 Colonoscopy report and pathology report (if biopsies are obtained) must be available in the source document:  • If the subject is ≥50 years of age, a colonoscopy within 10 years of the screening visit is required to exclude adenomatous polyps. Subjects whose adenomas have been completely excised at baseline will be eligible.  • If the subject has extensive colitis for ≥8 years or disease limited to left side of colon (ie, distal to splenic flexure) for ≥10 years, regardless of age, a colonoscopy within 1 year of the screening visit is required to survey for dysplasia. Subjects with dysplasia or cancer identified on biopsies will be excluded.  8. Subjects who have had surgery for UC or in the opinion of the Investigator, are likely to require surgery for UC during the study period.  9. Subjects who have positive stool examinations for enteric pathogens, pathogenic ova or parasites, or Clostridium difficile toxin at screening.  10. Subjects with clinically significant infections currently or within 6 months of baseline (eg, those requiring hospitalization or parenteral antimicrobial therapy or opportunistic infections), a history of any infection requiring antimicrobial therapy within 2 weeks of baseline, or a history of any infection otherwise judged by the investigator to have the potential for exacerbation by participation in the study.  11. Subjects with a history of more than one episode of herpes zoster, a history of disseminated herpes zoster or disseminated herpes simplex.  12. Subjects infected with human immunodeficiency virus (HIV) or hepatitis B or C viruses (Subjects with negative HBV surface antigen but positive HBV core antibody must have further testing for HBV surface antibody and if negative for HBV surface antibody, will be excluded from study enrollment).  13. Subjects who have been vaccinated with live or attenuated vaccine within 6 weeks of baseline or scheduled to receive these vaccines during study period or within 6 weeks after last dose of study medication.  14. Subjects with history of any lymphoproliferative disorder (such as EBV-related lymphoproliferative disorder, as reported in some subjects on other immunosuppressive drugs), history of lymphoma, leukemia, myeloproliferative disorders, multiple myeloma, or signs and symptoms suggestive of current lymphatic disease.  15. Subjects with malignancies or a history of malignancies, with the exception of adequately treated or excised non-metastatic basal cell or squamous cell cancer of the skin.  16. Subjects receiving prohibited concomitant medications, including moderate to potent CYP3A inducers or inhibitors in the specified time periods prior to the first dose of study drug or are expected to receive any of these medications during the study period.  17. Subjects with a history of bowel surgery within 6 months prior to baseline.  18. Subjects with significant trauma or major surgery within 4 weeks of screening visit.  19. Subjects likely to require any type of surgery during the study period.  20. Subjects with the following laboratory values at screening:  • Hemoglobin levels <9.0 g/dL or hematocrit <30%  • An absolute white blood cell (WBC) count of <3.0 x 10^9^ /L (<3000/mm^3^) or absolute neutrophil count of <1.2 x 10^9^/L (<1200/mm^3^).  • Thrombocytopenia, as defined by a platelet count <100 x 10^9^/L (<100,000/mm^3^).  • Subjects with estimated GFR ≤50 ml/min based on Cockcroft-Gault calculation.  • Subjects with total bilirubin, AST or ALT more than 1.5 times the upper limit of normal.  21. Subjects with evidence of or suspected liver disease ie, liver injury due to methotrexate or primary sclerosing cholangitis.  22. Subjects with current or recent history of severe, progressive, or uncontrolled renal, hepatic, hematological, gastrointestinal, metabolic (including uncontrolled hypercholesterolemia), endocrine, pulmonary, cardiac, neurological disease.  23. Subjects previously receiving either lymphocyte apheresis or selective monocyte granulocyte apheresis (eg, Cellsorba) within 1 year prior to baseline and throughout the study.  24. Subjects with any condition possibly affecting oral drug absorption (eg, gastrectomy, clinically significant diabetic gastroenteropathy, or certain types of bariatric surgery such as gastric bypass). Procedures such as gastric banding that simply divide the stomach into separate chambers are NOT exclusionary.  25. Women who are pregnant or lactating, or planning to become pregnant during the study period.  26. History of alcohol or drug abuse with less than 6 months of abstinence prior to baseline.  27. Screening 12-lead ECG that demonstrates clinically relevant abnormalities which may affect subject safety or interpretation of study results (ie, baseline QTcF >450 ms, complete LBBB, acute or indeterminate age myocardial infarction, 2nd-3rd degree AV block, or serious bradyarrhythmias or tachyarrhythmias; see (Appendix 4).  28. Donation of blood in excess of 500 mL within 8 weeks prior to baseline.  29. Subjects with a first-degree relative with a hereditary immunodeficiency.  30. Subjects who have previously participated in any study of investigational drug.  31. Subjects who have received any investigational drug or device within 3 months prior to baseline.  32. Subjects who, in the opinion of the investigator or Pfizer, will be uncooperative or unable to comply with study procedures.  33. Any other severe acute or chronic medical or psychiatric condition or laboratory abnormality that may increase the risk associated with study participation or investigational product administration or may interfere with the interpretation of study results and, in the judgment of the investigator, would make the subject inappropriate for entry into this study.  34. Subjects who are investigational site staff members or relatives of those site staff members or subjects who are Pfizer employees directly involved in the conduct of the trial. \|   Vedolizumab   \| **Inclusion Criteria (must meet all of the following to be eligible):** \| **Exclusion Criteria (meet any of the criteria will not be eligible):** \| \| --- \| --- \| \| 1. Age 18 to 80  2. Male or female patient who is voluntarily able to give informed consent 3. Female patients who:  • Are post-menopausal for at least 1 year before the screening visit, OR  • Are surgically sterile, OR  • If they are of childbearing potential, agree to practice 2 effective methods of contraception, at the same time, from four weeks before the first dose of study drug through 6 months after the last dose of study drug, OR agree to completely abstain from heterosexual intercourse.  Male patients, even if surgically sterilized (ie, status post-vasectomy), who:  • Agree to practice effective barrier contraception during the entire study treatment period and through 6 months after the last dose of study drug, OR  • Agree to completely abstain from heterosexual intercourse.  4. Diagnosis of ulcerative colitis established at least 6 months prior to enrollment by clinical and endoscopic evidence and corroborated by a histopathology report.  5. Moderately to severely active ulcerative colitis as determined by a Mayo score of 6 to 12 with an endoscopic subscore ≥2 within 7 days prior to the first dose of study drug  6. Evidence of ulcerative colitis extending proximal to the rectum (≥15 cm of involved colon)  7. Patients with extensive colitis or pancolitis of >8 years duration or left-sided colitis of >12 years duration must have documented evidence that a surveillance colonoscopy was performed within 12 months of the initial screening visit (may be performed during screening).  8. Patients with a family history of colorectal cancer, personal history of increased colorectal cancer risk, age >50 years, or other known risk factor must be up-to-date on colorectal cancer surveillance (may be performed during screening)  9. Demonstrated, over the previous 5 year period, an inadequate response to, loss of response to, or intolerance of at least one of the following agents as defined below:  • Corticosteroids  o Signs and symptoms of persistently active disease despite a history of at least one 4-week induction regimen that included a dose equivalent to prednisone 30 mg daily orally for 2 weeks or intravenously for 1 week OR  o Two failed attempts to taper corticosteroids to below a dose equivalent to prednisone 10 mg daily orally OR  o History of intolerance of corticosteroids (including, but not limited to Cushing’s syndrome, osteopenia/osteoporosis, hyperglycemia, insomnia, infection)  • Immunomodulators  o Signs and symptoms of persistently active disease despite a history of at least one 8 week regimen of oral azathioprine (≥1.5 mg/kg) or 6- mercaptopurine mg/kg (≥0.75 mg/kg) OR  o History of intolerance of at least one immunomodulator (including, but not limited to nausea/vomiting, abdominal pain, pancreatitis, LFT abnormalities, lymphopenia, TPMT genetic mutation, infection)  • TNFα antagonists  o Signs and symptoms of persistently active disease despite a history of at least one 4 week induction regimen of infliximab 5 mg/kg IV, 2 doses at least 2 weeks apart OR  o Recurrence of symptoms during maintenance dosing following prior clinical benefit (discontinuation despite clinical benefit does not qualify) OR  o History of intolerance of infliximab (including, but not limited to infusionrelated reaction, demyelination, congestive heart failure, infection)  10. May be receiving a therapeutic dose of the following drugs:  a. Oral 5-ASA compounds provided that the dose has been stable for the 2 weeks immediately prior to enrollment  b. Oral corticosteroid therapy (prednisone at a stable dose ≤30 mg/day, or equivalent steroid) provided that the dose has been stable for the 4 weeks immediately prior to enrollment if corticosteroids have just been initiated, or for the 2 weeks immediately prior to enrollment if corticosteroids are being tapered  c. Probiotics (eg, Culturelle, Saccharomyces boulardii) provided that the dose has been stable for the 2 weeks immediately prior to enrollment  d. Antidiarrheals (eg, loperamide, diphenoxylate with atropine) for control of chronic diarrhea  e. Azathioprine or 6-mercaptopurine provided that the dose has been stable for the 8 weeks immediately prior to enrollment \| **Gastrointestinal Exclusion Criteria**  1. Evidence of abdominal abscess or toxic megacolon at the initial screening visit  2. Extensive colonic resection, subtotal or total colectomy  3. Ileostomy, colostomy, or known fixed symptomatic stenosis of the intestine  4. Within 30 days prior to enrollment, have received any of the following for the treatment of underlying disease: a. Non-biologic therapies (eg, cyclosporine, thalidomide) b. A non-biologic investigational therapy c. An approved non-biologic therapy in an investigational protocol  5. Within 90 days prior to enrollment, have received any of the following:  a. Infliximab b. Other investigational or approved biologic agent  6. Any prior exposure to natalizumab or rituximab  7. Use of topical (rectal) treatment with 5-ASA or corticosteroid enemas/suppositories within 2 weeks of the administration of the first dose of study drug  8. Evidence of or treatment for C. difficile infection within 60 days or other intestinal pathogen within 30 days prior to enrollment  9. Currently require or are anticipated to require surgical intervention for UC during the study  10. History or evidence of adenomatous colonic polyps that have not been removed  11. History or evidence of colonic mucosal dysplasia  12. Diagnosis of Crohn’s colitis or indeterminate colitis  **Infectious Disease Exclusion Criteria**  1. Chronic hepatitis B or C infection  2. Active or latent tuberculosis, regardless of treatment history, as evidenced by any of the following:  a. History of tuberculosis  b. A positive diagnostic tuberculosis (TB) test within one month of enrollment defined as:  i. a positive QuantiFERON test or 2 successive indeterminate QuantiFERON tests OR  ii. a tuberculin skin test reaction ≥10 mm ( ≥5 mm in patients receiving the equivalent of > 15 mg/day prednisone).  c. Chest X-ray within 3 months of enrollment in which active or latent pulmonary tuberculosis cannot be excluded  3. Any identified congenital or acquired immunodeficiency (eg, common variable immunodeficiency, human immunodeficiency virus [HIV] infection, organ transplantation)  4. Any live vaccinations within 30 days prior to study drug administration except for the influenza vaccine  5. Clinically significant extra-intestinal infection (eg, pneumonia, pyelonephritis) within 30 days prior to enrollment  **General Exclusion Criteria**  1. Previous exposure to MLN0002  2. Female patients who are lactating or have a positive serum pregnancy test during the screening period or a positive urine pregnancy test on Day 1 prior to study drug administration.  3. Any unstable or uncontrolled cardiovascular, pulmonary, hepatic, renal, gastrointestinal, genitourinary, hematological, coagulation, immunological, endocrine/metabolic, or other medical disorder that, in the opinion of the investigator, would confound the study results or compromise patient safety  4. Had any surgical procedure requiring general anesthesia within 30 days prior to enrollment or is planning to undergo major surgery during the study period 5. Any history of malignancy, except for the following:  (a) adequately-treated non-metastatic basal cell skin cancer;  (b) any other type of non-melanoma skin cancer that has been adequately treated and has not recurred for at least 1 year prior to enrollment; and  (c) adequately treated in situ cervical cancer that has not recurred for at least 1 year prior to enrollment  6. History of any major neurological disorders, including stroke, multiple sclerosis, brain tumor, or neurodegenerative disease  7. Positive PML subjective symptom checklist prior to the administration of the first dose of study drug  8. Any of the following laboratory abnormalities during the screening period  a. Hemoglobin level <8g/dL  b. WBC count < 3 × 10^9^ /L  c. Lymphocyte count <0.5 x 10^9^/L  d. Platelet count <100 x 10^9^/L or >1200 × 10^9^/L  e. Alanine aminotransferase (ALT) or aspartate aminotransferase (AST) >3 × the upper limit of normal (ULN)  f. Alkaline phosphatase >3 × ULN  g. Serum creatinine >2 × ULN  9. Current or recent history (within one year prior to enrollment) of alcohol dependence or illicit drug use  10. Active psychiatric problems that, in the investigator’s opinion, may interfere with compliance with the study procedures  11. Unable to attend all the study visits or comply with study procedures \|   Ustekinumab:   \| **Inclusion Criteria (must meet all of the following to be eligible):** \| **Exclusion Criteria (meet any of the criteria will not be eligible):** \| \| --- \| --- \| \| 1. Be a man or woman 18 years of age or older.  2. Has a clinical diagnosis of UC at least 3 months before screening.  3. Has moderately to severely active UC, defined as a baseline (Week 0) Mayo score of 6 to 12, inclusive, using the Mayo endoscopy subscore assigned during the central reading of the video endoscopy.  4. Has a screening endoscopy with ≥2 endoscopy subscore of the Mayo score as determined by a central reading of the video endoscopy.  5. Have failed biologic therapy, ie, have received treatment with 1 or more TNF antagonists or vedolizumab at a dose approved for the treatment of UC, and have a documented history of failure to respond to or tolerate such treatment  OR  Be naïve to biologic therapy (ie, TNF antagonists or vedolizumab) or not have demonstrated a history of failure to respond to, or tolerate, a biologic therapy and have a prior or current UC medication history that includes at least 1 of the following:  a. Inadequate response to or failure to tolerate current treatment with oral corticosteroids or immunomodulators (6-MP or AZA), OR  b. History of failure to respond to, or tolerate, at least 1 of the following therapies: oral or IV corticosteroids or immunomodulators (6-MP or AZA) (Attachment 2), OR  c. History of corticosteroid dependence (ie, an inability to successfully taper corticosteroids without a return of the symptoms of UC; Attachment 2).  6. Before the first administration of study agent, the following conditions must be met:  a. If receiving conventional immunomodulators (ie, AZA, 6-MP, or MTX), must have been taking them for ≥12 weeks, and on a stable dose for at least 4 weeks.  b. If AZA, 6-MP, or MTX has been recently discontinued, it must have been stopped for at least 4 weeks.  c. If receiving oral 5-ASA compounds, the dose must have been stable for at least 2 weeks.  d. If receiving oral corticosteroids, the dose must be ≤20 mg/day prednisone or its equivalent and must have been stable for at least 2 weeks.  e. If receiving budesonide, the dose must have been stable for at least 2 weeks. f. If oral 5-ASA compounds or oral corticosteroids (including budesonide) have been recently discontinued, they must have been stopped for at least 2 weeks.  7. The following medications/therapies must have been discontinued before first administration of study agent:  a. Vedolizumab for at least 4 months.  b. TNF-antagonist therapy (eg, infliximab, etanercept, certolizumab, adalimumab, golimumab) for at least 8 weeks.  c. Cyclosporine, tacrolimus, or sirolimus, for at least 4 weeks.  d. 6-thioguanine (6-TG) must have been discontinued for at least 4 weeks.  e. Rectal corticosteroids (ie, corticosteroids [including budesonide] administered to the rectum or sigmoid colon via foam or enema or suppository) for at least 2 weeks.  f. Rectal 5-ASA compounds (ie, 5-ASAs administered to the rectum or sigmoid colon via foam or enema or suppository) for at least 2 weeks.  g. Parenteral corticosteroids for at least 2 weeks.  h. Total parenteral nutrition (TPN) for at least 2 weeks.  i. Antibiotics for the treatment of UC (eg, ciprofloxacin, metronidazole, or rifaximin) for at least 2 weeks.  8. A subject ≥45 years of age must either have had a colonoscopy to assess for the presence of adenomatous polyps within 5 years before the first administration of study agent or a colonoscopy to assess for the presence of adenomatous polyps at the screening visit. The adenomatous polyps must be removed before the first administration of study agent.  9. A subject who has had extensive colitis for ≥8 years, or disease limited to the left side of the colon for ≥10 years, must either have had a colonoscopy to assess for the presence of dysplasia within 1 year before the first administration of study agent or a colonoscopy to assess for the presence of malignancy at the screening visit. 10. Is considered eligible according to the following tuberculosis (TB) screening criteria:  a. Has no history of latent or active TB before screening. An exception is made for subjects who have a history of latent TB and are currently receiving treatment for latent TB, will initiate treatment for latent TB before the first administration of study agent, or have documentation of having completed appropriate treatment for latent TB within 3 years before the first administration of study agent. It is the responsibility of the investigator to verify the adequacy of previous anti-tuberculous treatment and provide appropriate documentation.  b. Has no signs or symptoms suggestive of active TB upon medical history and/or physical examination.  c. Has had no recent close contact with a person with active TB or, if there has been such contact, will be referred to a physician specializing in TB to undergo additional evaluation and, if warranted, receive appropriate treatment for latent TB before the first administration of study agent.  d. Within 2 months before the first administration of study agent, has a negative QuantiFERON-TB Gold test result, or has a newly identified positive QuantiFERON-TB Gold test result in which active TB has been ruled out and for which appropriate treatment for latent TB has been initiated before the first administration of study agent. Within 2 months before the first administration of study agent, a negative tuberculin skin test, or a newly identified positive tuberculin skin test in which active TB has been ruled out and for which appropriate treatment for latent TB has been initiated before the first administration of study agent, is additionally required if the QuantiFERON-TB Gold test is not approved/registered in that country or the tuberculin skin test is mandated by local health authorities.  e. Has a chest radiograph (posterior-anterior view), taken within 3 months before the first administration of study agent and read by a qualified radiologist, with no evidence of current, active TB or old, inactive TB  11. Before randomization, a woman must be either:  a. Not of childbearing potential: premenarchal; postmenopausal (>45 years of age with amenorrhea for at least 12 months or any age with amenorrhea for at least 6 months and a serum follicle-stimulating hormone level >40 IU/L); permanently sterilized (eg, bilateral tubal occlusion [which includes tubal ligation procedures consistent with local regulations], hysterectomy, bilateral salpingectomy, bilateral oophorectomy); or otherwise be incapable of pregnancy.  OR  b. Of childbearing potential: if heterosexually active, must be practicing a highly effective method of birth control consistent with local regulations regarding the use of birth control methods for subjects participating in clinical studies: eg, established use of oral, injected or implanted hormonal methods of contraception; placement of an intrauterine device (IUD) or intrauterine system (IUS); barrier methods: condom with spermicidal foam/gel/film/cream/suppository or occlusive cap (diaphragm or cervical/vault caps) with spermicidal foam/gel/film/cream/suppository; male partner sterilization (the vasectomized partner should be the sole partner for that subject); true abstinence (when this is in line with the preferred and usual lifestyle of the subject).  12. A woman of childbearing potential must have a negative serum (β-human chorionic gonadotropin) pregnancy test result at screening and a negative urine pregnancy test result at Week 0. 13. A woman must agree not to donate eggs (ova, oocytes) for the purposes of assisted reproduction during the study and for 20 weeks after the last study agent administration.  14. A man who is sexually active with a woman of childbearing potential and who has not had a vasectomy must agree to use a barrier method of birth control, eg, either condom with spermicidal foam/gel/film/cream/suppository or partner with occlusive cap (diaphragm or cervical/vault caps) with spermicidal foam/gel/film/cream/suppository; all men must also agree not to donate sperm during the study and for 20 weeks after receiving the last administration of study agent.  15. Has screening laboratory test results within the following parameters: a. Hemoglobin ≥8.0 g/dL b. White blood cell count (WBC) ≥2.5 × 103 /µL c. Neutrophils ≥1.5 × 103 /µL d. Platelets ≥100 × 103 /µL e. Serum creatinine  f. Alanine aminotransferase (ALT) and aspartate aminotransferase (AST) concentrations must be within 2 times the upper limit of the normal range (ULN) for the laboratory conducting the test.  16. Be willing and able to adhere to the prohibitions and restrictions specified in this protocol.  17. Each subject must sign an informed consent form (ICF) indicating that he or she understands the purpose of and procedures required for the study and is willing to participate in the study. In regions where the legal age of consent is older than 18 years, informed consent must be obtained from and signed by both the subject and his or her legally acceptable representative.  18. Each subject must sign a separate ICF if he or she agrees to provide optional DNA samples for research where local regulations permit. (In regions where the legal age of consent is older than 18 years, informed consent must be obtained from and signed by both the subject and his or her legally acceptable representative.) Refusal to give consent for the optional DNA samples does not exclude a subject from participation in the study. \| 1. Has severe extensive colitis as evidenced by:  a. Current hospitalization for the treatment of UC, OR  b. Investigator judgment that the subject is likely to require a colectomy within 12 weeks of baseline, OR  c. Symptom complex at screening or baseline visits that includes at least 4 of the following:  1) Diarrhea with ≥6 bowel movements/day with macroscopic blood in stool  2) Focal severe or rebound abdominal tenderness  3) Persistent fever (≥37.5°C)  4) Tachycardia (>90 beats/minute)  5) Anemia (hemoglobin <8.5 g/dL)  2. Has UC limited to the rectum only or to <20 cm of the colon.  3. Presence of a stoma.  4. Presence or history of a fistula.  5. Require, or required within the 2 months before screening, surgery for active gastrointestinal bleeding, peritonitis, intestinal obstruction, or intra-abdominal or pancreatic abscess requiring surgical drainage, or other conditions possibly confounding the evaluation of benefit from study agent treatment.  6. Presence of symptomatic colonic or small bowel obstruction, confirmed by objective radiographic or endoscopic evidence of a stricture with resulting obstruction (dilation of the colon or small bowel proximal to the stricture on barium radiograph or an inability to traverse the stricture at endoscopy).  7. History of extensive colonic resection (eg, less than 30 cm of colon remaining) that would prevent adequate evaluation of the effect of study agent on clinical disease activity.  8. History of colonic mucosal dysplasia. Subjects will not be excluded from the study because of a pathology finding of “indefinite dysplasia with reactive atypia.”  9. Presence on screening endoscopy of adenomatous colonic polyps, if not removed before study entry, or history of adenomatous colonic polyps that were not removed.  10. Diagnosis of indeterminate colitis, microscopic colitis, ischemic colitis, or Crohn’s disease or clinical findings suggestive of Crohn’s disease.  11. Has a stool culture or other examination positive for an enteric pathogen, including Clostridium difficile toxin, in the previous 4 months, unless a repeat examination is negative and there are no signs of ongoing infection with that pathogen.  Concomitant or previous medical therapies received:  12. Has received the following concomitant or previous medical therapies:  a. A biologic therapy targeted at IL-12 and/or IL-23 (eg, ustekinumab, briakinumab, guselkumab).  b. Natalizumab within 12 months of first study agent administration.  c. Agents that deplete B or T cells (eg, rituximab, alemtuzumab) within 12 months of first study agent administration, or continue to manifest depletion of B or T cells more than 12 months after completion of therapy with lymphocyte-depleting agents.  d. Any investigational drug within 4 weeks before first administration of study agent or within 5 half-lives of the investigational agent, whichever is longer.  e. Apheresis (ie, Adacolumn apheresis) within 2 weeks before first administration of study agent.  Infections or predisposition to infections:  13. Has a history of latent or active granulomatous infection, including histoplasmosis or coccidioidomycosis, before screening.  14. Has a history of, or ongoing, chronic or recurrent infectious disease, including but not limited to, chronic renal infection, chronic chest infection (eg, bronchiectasis), sinusitis, recurrent urinary tract infection (eg, recurrent pyelonephritis, recurrent cystitis), an open, draining, or infected skin wound, or an ulcer.  15. Has a chest radiograph within 3 months before the first administration of study agent that shows an abnormality suggestive of a malignancy or current active infection, including TB.  16. Have a history of human immunodeficiency virus (HIV) antibody positive, or tests positive for HIV at screening.  17. Are seropositive for antibodies to hepatitis C virus (HCV). 18. Subjects must undergo screening for hepatitis B virus (HBV). At a minimum, this includes testing for HBV surface antigen (HBsAg), HBV surface antibody (anti-HBs), and HBV core antibody (anti-HBc) total:  a. Subjects who test negative for all HBV screening tests (ie, HBsAg-, anti-HBc-, and antiHBs-) are eligible for this study.  b. Subjects who test positive for surface antigen (HBsAg+) are not eligible for this study, regardless of the results of other hepatitis B tests.  c. Subjects who test negative for surface antigen (HBsAg-) and test positive for core antibody (anti-HBc+) and surface antibody (anti-HBs+) are eligible for this study.  d. Subjects who test positive only for surface antibody (anti-HBs+) are eligible for this study.  e. Subjects who test positive only for core antibody (anti-HBc+) must undergo further testing for hepatitis B deoxyribonucleic acid (HBV DNA test). If the HBV DNA test is positive, the subject is not eligible for this study. If the HBV DNA test is negative, the subject is eligible for this study. In the event the HBV DNA test cannot be performed, the subject is not eligible for this study.  19. Has had a Bacille Calmette-Guerin (BCG) vaccination within 12 months or any other live bacterial or live viral vaccination within 12 weeks before baseline.  20. Has or has ever had a nontuberculous mycobacterial infection or serious opportunistic infection (eg, cytomegalovirus colitis, Pneumocystis carinii, aspergillosis).  21. Has had a serious infection (eg, hepatitis, pneumonia, or pyelonephritis), has been hospitalized for an infection, or has been treated with parenteral antibiotics for an infection within 2 months before first administration of study agent. Less serious infections (eg, acute upper respiratory tract infection, simple urinary tract infection) need not be considered exclusionary at the discretion of the investigator.  22. Has evidence of a herpes zoster infection ≤8 weeks before baseline.  Malignancy or increased potential for malignancy:  23. Has any known malignancy or has a history of malignancy (with the exception of basal cell carcinoma; squamous cell carcinoma in situ of the skin; or cervical carcinoma in situ that has been treated with no evidence of recurrence; or squamous cell carcinoma of the skin that has been treated with no evidence of recurrence within 5 years before screening).  24. Presence or history of lymphoproliferative disease including lymphoma, or signs and symptoms suggestive of possible lymphoproliferative disease, such as lymphadenopathy of unusual size or location (eg, nodes in the posterior triangle of the neck, infraclavicular, epitrochlear, or periaortic areas), or clinically significant hepatomegaly or splenomegaly, or monoclonal gammopathy of undetermined significance.  **Coexisting medical conditions or past medical history:**  25. Has known allergies, hypersensitivity, or intolerance to ustekinumab or its excipients (refer to the ustekinumab IB).  26. Has severe, progressive, or uncontrolled renal, hepatic, hematologic, endocrine, pulmonary, cardiac, neurologic, psychiatric, or cerebral disease, or signs or symptoms thereof.  27. Has a transplanted organ (with the exception of a corneal transplant performed >12 weeks before screening).  28. Has previously undergone allergy immunotherapy for prevention of anaphylactic reactions.  29. Has a history of drug or alcohol abuse according to the Diagnostic and Statistical Manual of Mental Disorders, 4th edition (DSM-IV), within 1 year before screening.  30. Has poor tolerability of venipuncture or lacks adequate venous access for required blood sample collections during the study period.  31. Is a woman who is pregnant, or breast-feeding, or planning to become pregnant, or is a man who plans to father a child while enrolled in this study or within 20 weeks after the last dose of study agent.  32. Has any condition for which, in the opinion of the investigator, participation would not be in the best interest of the subject (eg, compromise the well-being) or that could prevent, limit, or confound the protocol-specified assessments.  **General:**  33. Is currently participating or intends to participate in any other study using an investigational agent or procedure during participation in this study.  34. Employees of the investigator or study site with direct involvement in the proposed study or other studies under the direction of that investigator or study site, as well as family members of the employees or the investigator. \| |
| --- | --- | --- | --- | --- | --- | --- | --- | --- | --- | --- | --- | --- |

| Etrasimod (APD334) APD334-301:   \| **Inclusion Criteria (must meet all of the following to be eligible):** \| **Exclusion Criteria (meet any of the criteria will not be eligible):** \| \| --- \| --- \| \| 1. Men or women 16 to 80 years of age, inclusive, at the time of assent/consent  2. Ability to provide written informed consent or assent (parent or legal guardian must provide consent for a subject < 18 years of age who has assented to participate in the study or as required per local regulations) and to be compliant with the schedule of protocol assessments  **Disease-specific inclusion criteria**  3. Diagnosed with UC ≥ 3 months prior to screening. The diagnosis of UC must be confirmed by endoscopic and histologic evidence. The endoscopy and histology report should be present in the source documents; however, if not available, the screening endoscopy and histology may serve as such  4. Active UC confirmed by endoscopy with ≥ 10 cm rectal involvement. Inclusion of subjects with proctitis only at baseline will be capped at 15% of the total subjects enrolled.  5. Moderately to severely active UC defined as MMS of 4 to 9, including an ES of ≥ 2 and RB score ≥ 1  6. Received a surveillance colonoscopy (performed according to local standard) within 12 months before baseline to rule out dysplasia in subjects with pancolitis > 8 years duration or subjects with left-sided colitis > 12 years duration. Subjects without a surveillance colonoscopy within the prior 12 months will have a colonoscopy at screening (ie, in place of screening proctosigmoidoscopy). Any adenomatous polyps must be removed prior to their first dose of study treatment  **Prior treatment:**  7. Demonstrated an inadequate response to, loss of response to, or intolerance to at least 1 of the following therapies as defined below:  **Conventional therapy**   1. Oral 5-aminosalicylic acid (5-ASA) compounds 2. Corticosteroids 3. Thiopurines   **Biologic therapy or JAK inhibitor therapy**   1. Antitumor necrosis factor alpha (TNFα) antibodies (eg. infliximab, adalimumab, golimumab, or biosimilars) 2. Anti-integrin antibodies (eg. vedolizumab) 3. JAK inhibitors (eg, tofacitinib)   Note: The medication used to qualify the subject for entry into this category must be approved for the treatment of UC in the country of use.  Inadequate response, loss of response, and intolerance are defined as:   - Inadequate response: Signs and symptoms of persistently active disease despite a history of completing a regimen at doses per the current labeling and/or institutional standard of care - Loss of response: Recurrence of symptoms of active disease during treatment following prior clinical benefit (discontinuation despite clinical benefit does not qualify as having failed or being intolerant to UC biologic therapy) - Intolerance: Including, but not limited to infusion- or injection-related reaction, demyelination, congestive heart failure, infection, or any other related adverse event that led to a reduction in dose or discontinuation of the medication   **Concomitant treatments**  8. Subjects are permitted to be receiving a therapeutic dose of the following drugs:   - Oral 5-ASA compounds provided the dose has been stable for ≥ 2 weeks immediately prior to randomization - Oral corticosteroid therapy (prednisone at a stable dose ≤ 20 mg/day, budesonide at a stable dose ≤ 9 mg/day, or equivalent steroid) provided the dose has been stable for the 4 weeks immediately prior to the screening endoscopy assessment (Note: Subjects on existing oral corticosteroid therapy will be tapered during the 40-Week Treatment Period.) - Immunosuppressive agents such as oral azathioprine (AZA) or 6-mercaptopurine (6-MP) **must be discontinued ≥ 2 weeks prior to randomization** - Probiotics (eg. Culturelle®, *Saccharomyces boulardii*) provided the dose has been stable for the 2 weeks immediately prior to randomization - Antidiarrheals (eg. loperamide, diphenoxylate with atropine) for control of chronic diarrhea   If oral aminosalicylates or corticosteroids have been recently discontinued, they must have been stopped for at least 2 weeks prior to the endoscopy used for the baseline MMS  **Other general inclusion criteria**  9. Vital signs at screening and pre-randomization taken in the sitting position: heart rate ≥ 50 bpm, systolic blood pressure (BP) ≥ 90 mm Hg, and diastolic BP ≥ 55 mm Hg  10. Screening and prerandomization 12-lead ECG showing no clinically significant abnormalities with a PR interval ≤ 200 ms, Fridericia’s corrected QT interval (QTcF) < 450 ms (men) or QTcF < 470 ms (women)  11. Adequate hematological function defined by white blood cell count ≥ 3.5 × 109/L with absolute neutrophil count (ANC) ≥ 1.5 × 109/L, lymphocyte count ≥ 0.8 × 109/L, platelet count ≥ 100 × 109/L, and hemoglobin ≥ 8 g/dL  12. Adequate hepatic function defined by a total bilirubin level ≤ 1.5 × the upper limit of normal (ULN) range and aspartate aminotransferase (AST) and alanine aminotransferase (ALT) levels ≤ 3.0 × ULN. Subjects with an isolated total bilirubin and normal AST and ALT diagnosed with Gilbert’s syndrome may participate  13. Adequate renal function defined by an estimated glomerular filtration rate ≥ 30 mL/min/1.73 m2 by the CKD-EPI equation at screening  14. Eligible women of childbearing potential must be:   1. Nonpregnant, evidenced by a negative serum beta-human chorionic gonadotropin (β-hCG) pregnancy test at screening and a urine dipstick pregnancy test at Day 1 2. Not breastfeeding   15. Both men and women subjects agree to use a highly effective method of birth control throughout the entire study period, from informed consent through the adverse event reporting period (30 days after the last dose of study treatment), if the possibility of conception exists. Eligible men and women subjects must also agree not to participate in a conception process (ie. actively attempt to become pregnant or to impregnate, sperm donation, in vitro fertilization) during the study and for 30 days after the last dose of study treatment. Highly effective birth control methods include the following:   - Oral, implantable, or injectable contraceptives (starting ≥ 60 days before dosing) in combination with a diaphragm with vaginal spermicide, cervical cap with vaginal spermicide, or male condom; hormonal contraceptives (subjects should be consistently taking the hormonal contraceptive for at least 3 months [90 days] prior to screening) - Standard intrauterine device (IUD; eg. Copper T 380A IUD), intrauterine system (IUS; eg. LNg 20 IUS - progesterone IUD), progesterone implant, or tubal sterilization (≥ 180 days after surgery) - Vasectomized male subjects using a condom, partner using diaphragm with spermicide, cervical cap with spermicide, estrogen and progesterone oral contraceptives (“the pill”), estrogen and progesterone transdermal patch, vaginal ring, or progesterone injection - Complete sexual abstinence defined as refraining from heterosexual intercourse for the entire period of risk associated with study treatments. The reliability of sexual abstinence needs to be evaluated in relation to the duration of the clinical study and the preferred and usual lifestyle of the subject. Periodic abstinence (calendar, symptothermal, post-ovulation methods) is not acceptable   Note: Women who are surgically sterile or postmenopausal (defined as: 12 consecutive months with no menses without an alternative medical cause) are not considered to be of childbearing potential. If of childbearing potential, female partners of male subjects should agree to utilize a highly effective method of contraception for the duration of study participation \| **Exclusions related to general health**  1. Severe extensive colitis as evidenced by: Physician judgment that the subject is likely to require hospitalization for medical care or surgical intervention of any kind for UC (eg. colectomy) within 12 weeks of baseline   - Current evidence of fulminant colitis, toxic megacolon or recent history (within last 6 months) of toxic megacolon, or bowel perforation - Previous total or partial colectomy   2. Diagnosis of CD or indeterminate colitis or the presence or history of a fistula consistent with CD  3. Diagnosis of microscopic colitis, ischemic colitis, or infectious colitis  4. Hospitalization for exacerbation of UC requiring intravenous (IV) steroids within 12 weeks of screening (a single dose of IV steroids given is acceptable)  5. Positive assay or stool culture for pathogens (ova and parasite examination, bacteria) or positive test for *Clostridium difficile* toxin at screening (If *C. difficile* is positive, the subject may be treated and retested ≥ 4 weeks after completing treatment)  6. Pregnancy, lactation, or a positive serum β-hCG measured during screening  7. Clinically relevant hematologic, hepatic, neurological, pulmonary, ophthalmological, endocrine, metabolic (including, but not limited to, hypo- and hyperkalemia), psychiatric, or other major systemic disease making implementation of the protocol or interpretation of the study difficult or would put the subject at risk  8. Recent history (within 2 months of the Screening Visit) of cardiovascular disease, including myocardial infarction or unstable angina  9. Any history of the following, unless treated with an implanted pacemaker or an implanted cardioverter-defibrillator with pacing:   - History or presence of symptomatic bradycardia - History of sick sinus syndrome or neurocardiogenic syncope - Second or third-degree AV block - Periods of asystole > 3 seconds   10. Forced expiratory volume at 1 second (FEV1) or forced vital capacity (FVC) < 70% of predicted values and FEV1/FVC ratio < 0.70 at screening  11. Uncontrolled diabetes as determined by hemoglobin A1c (HbA1c) > 9% at screening, or subjects with diabetes with significant comorbid conditions such as retinopathy  12. History of macular edema or retinopathy  13. Current or past history of active tuberculosis (TB), history of untreated latent TB infection, or test positive for latent TB infection at screening (refer to Appendix 2 for details on TB screening requirements and interpretation of test results). The following are EXCEPTIONS to this exclusion criteria:   - Subjects with latent TB, who have been ruled out for active TB, have completed an appropriate course of TB prophylaxis treatment per national/local medical guidelines or WHO guidelines, and have not had recent close contact with a person with active TB are eligible to enroll in the study. It is the responsibility of the Investigator to verify the adequacy of previous TB treatment and provide appropriate documentation - Subjects diagnosed with latent TB at screening, ruled out for active TB and received at least 4 weeks of an appropriate TB prophylaxis regimen may be rescreened for enrollment   Note: The 2 exceptions to this exclusion criterion outlined above do NOT apply to subjects in countries identified by WHO as a high multi-drug resistant TB burden country due to the high risk of latent infection with multi-drug resistance  14. Known active bacterial, viral, fungal, mycobacterial infection, or other infection (including TB or atypical mycobacterial disease) or any major episode of infection that required hospitalization or treatment with IV antibiotics within 30 days of screening or during screening or oral antibiotics within 14 days prior to screening. Fungal infection of nail beds is allowed  15. Have human immunodeficiency virus (HIV)/acquired immune deficiency syndrome or test positive for HIV antibodies at screening  16. Have acute or chronic hepatitis B infection or test positive for hepatitis B virus (HBV) at screening (positive for hepatitis B surface antigen [HBsAg], or negative for HBsAg and positive for antihepatitis B core antibody in conjunction with detectable HBV DNA, or detectable HBV DNA)  17. Have current hepatitis C infection or test positive for hepatitis C virus (HCV) at screening as defined by positive for hepatitis C antibody and detectable HCV RNA  18. History of an opportunistic infection (eg. pneumocystis carinii, cryptococcal meningitis, progressive multifocal leukoencephalopathy) or serious bacterial, viral, or fungal infections (eg. disseminated herpes simplex, disseminated herpes zoster) and requiring IV medication(s) ≤ 3 weeks prior to randomization  19. History of or currently active primary or secondary immunodeficiency  20. History of cancer within the last 5 years, including solid tumors and hematological malignancies (except basal cell and in situ squamous cell carcinomas of the skin that have been excised and resolved) or colonic mucosal dysplasia  21. History of lymphoproliferative disorder, lymphoma, leukemia, myeloproliferative disorder, or multiple myeloma  22. History of alcohol or drug abuse within 1 year prior to randomization  **Exclusions related to medications**  23. Prior treatment with S1P receptor modulators  24. Treatment with a biologic agent within 8 weeks or 5 elimination half-lives, whichever is shorter, prior to randomization  25. Treatment with an investigational therapy within 3 months prior to randomization  26. Treatment failure with ≥ 3 biologic agents or ≥ 2 biologics plus a JAK inhibitor approved for treatment of UC  27. Treatment with topical rectal 5-ASA, short-chain fatty acid enemas, or steroids within 2 weeks of screening or during screening  28. Treatment with cyclosporine, tacrolimus, sirolimus, methotrexate, or mycophenolate mofetil (MMF) within 16 weeks of screening  29. Receipt of a live vaccine within 4 weeks prior to randomization  30. Previous treatment with natalizumab  31. Previous treatment with lymphocyte-depleting therapies (eg. alemtuzumab, anti-CD4, cladribine, rituximab, ocrelizumab, cyclophosphamide, mitoxantrone, total body irradiation, bone marrow transplantation, alemtuzumab, daclizumab)  32. Previous treatment with D-penicillamine, leflunomide, or thalidomide  33. Treatment with IV immune globulin or plasmapheresis within 3 months prior to randomization  34. Chronic use of therapies that moderately/strongly inhibit/induce cytochrome P450 (CYP) 2C8 and 2C9 metabolism and inhibitors of UGT1A7 within 4 weeks prior to randomization \| |
| --- | --- | --- | --- | --- |

Mirikizumab (LY3074828):

| **Inclusion Criteria (patients with UC must meet all requirements to be eligible):** | **Exclusion Criteria (patients will be excluded if they meet any of the criteria):** |
| --- | --- |
| **Informed Consent**  1. Have given written informed consent approved by the ethical review board (ERB) governing this site  **Patient Characteristics**  2. Are male or female patients ≥ 18 and ≤ 80 years of age at the time of initial screening  2a. male patients:   - No male contraception required except in compliance with specific local government study requirements   2b. female patients:  **Women of childbearing potential:**   - must test negative for pregnancy prior to initiation of treatment as indicated by a negative serum pregnancy test at the screening visit followed by a negative urine pregnancy test within 24 hours prior to exposure   **AND**   - must agree to either remain abstinent, if complete abstinence is their preferred and usual lifestyle, or remain in same-sex relationships, if part of their preferred and usual lifestyle, without sexual relationships with males. Periodic abstinence (for example, calendar, ovulation, symptothermal, or post ovulation methods), declaration of abstinence just for the duration of a trial, and withdrawal are not acceptable methods of contraception   **OR**  must use 2 effective methods of contraception for the entirety of the study. Abstinence or contraception must continue following completion of study drug administration for 20 weeks   - two effective methods of contraception (such as male or female condoms with spermicide, diaphragms with spermicide, or cervical sponges) will be used. The subject may choose to use a double barrier method of contraception. Barrier protection methods without concomitant use of a spermicide are not a reliable or acceptable method. Thus, each barrier method must include use of a spermicide. It should be noted that the use of male and female condoms as a double barrier method is not considered acceptable because of the high failure rate when these methods are combined - of note, 1 of the 2 methods of contraception may be a highly effective (less than 1% failure rate) method of contraception (such as combination oral contraceptives, implanted contraceptives, or intrauterine devices)   **Women not of childbearing potential may participate and include those who are:**   - Infertile due to surgical sterilization (hysterectomy, bilateral oophorectomy, or tubal ligation), congenital anomaly such as mullerian agenesis; or - Postmenopausal – defined as either: - a woman at least 50 years of age with an intact uterus, not on hormone therapy, who has had either - cessation of menses for at least 1 year or - at least 6 months of spontaneous amenorrhea with a follicle-stimulating hormone (FSH) level >40 mIU/mL; or - a woman 55 years or older not on hormone therapy, who has had at least 6 months of spontaneous amenorrhea; or - a woman at least 55 years of age with a diagnosis of menopause prior to starting hormone replacement therapy   3. Venous access sufficient to allow blood sampling and IV administration as per the protocol  **Disease-Specific Inclusion Criteria**  4. Have had an established diagnosis of UC of ≥3 months in duration before baseline (Week 0), which includes endoscopic evidence of UC and a histopathology report that supports a diagnosis of UC (see Section 9.1.1.3). Supportive endoscopy and histopathology reports must be available in the source documents. Patients with rectal sparing on baseline endoscopy must have documentation of rectal involvement on a prior endoscopy and histopathology report to confirm UC diagnosis  5. Have moderately to severely active UC as defined by a modified Mayo score (MMS) of 4 to 9 with an endoscopic subscore (ES) ≥2, with endoscopy performed within 14 days before baseline  6. Have evidence of UC extending beyond the rectum (more proximal to the rectosigmoid junction). The rectosigmoid junction lies approximately 10 to 15 cm from the anal margin  7. Have documentation of:  7a. a surveillance colonoscopy (performed according to local standard) within 12 months before baseline for:   - patients with pancolitis of >8 years’ duration, or - patients with left-sided colitis of >12 years’ duration, or - patients with primary sclerosing cholangitis   **OR**  7b. in patients for whom Inclusion Criterion 7a does not apply, up-to-date colorectal cancer surveillance (performed according to local standard)  At the discretion of the investigator, a colonoscopy (instead of a flexible sigmoidoscopy) can be performed as the screening endoscopy for this study. Patients who do not have a colonoscopy report available in source documentation will have a colonoscopy at screening  **Prior Medication Failure Criteria**  8. Patients must have an inadequate response to, loss of response to, or intolerance to at least 1 of the medications described in Inclusion Criteria 8a OR 8b. Documentation of dose, frequency, route of administration and duration of the prior failed treatment is required  8a. Conventional-failed patients: Patients who have an inadequate response to, loss of response to, or are intolerant to at least one of the following medications:   - corticosteroids   - corticosteroid-refractory colitis, defined as signs and/or symptoms of active UC despite oral prednisone (or equivalent oral corticosteroid excluding budesonide MMX and beclomethasone dipropionate gastro- resistant prolonged-release tablet) at doses of at least 30 mg/day for a minimum of 2 weeks; or   - corticosteroid-dependent colitis, defined as:     1. an inability to reduce corticosteroids below the equivalent of prednisone 10 mg/day within 3 months of starting corticosteroids without a return of signs and/or symptoms of active UC; or     2. a relapse within 3 months of completing a course of corticosteroids; or   - history of intolerance of corticosteroids (including, but not limited to, Cushing’s syndrome, osteopenia/osteoporosis, hyperglycemia, or neuropsychiatric side-effects, including insomnia, associated with corticosteroid treatment) history of intolerance of corticosteroids (including, but not limited to, Cushing’s syndrome, osteopenia/osteoporosis, hyperglycemia, or neuropsychiatric side-effects, including insomnia, associated with corticosteroid treatment) - immunomodulators:   - signs and/or symptoms of persistently active disease despite at least 3 months’ treatment with one of the following:     1. oral AZA (≥1.5 mg/kg/day) or 6-MP (≥0.75 mg/kg/day), or     2. oral AZA or 6-MP within a therapeutic range as judged by thioguanine metabolite testing, or     3. a combination of a thiopurine and allopurinol within a therapeutic range as judged by thioguanine metabolite testing - history of intolerance to at least 1 immunomodulator (including but not limited to nausea/vomiting, abdominal pain, pancreatitis, liver function test abnormalities, and lymphopenia)   **AND**   - have neither failed nor demonstrated an intolerance to a biologic medication (anti-TNF antibody or anti-integrin antibody) that is indicated for the treatment of UC   8b. Biologic-failed patients: Patients who have an inadequate response to, loss of response to, or are intolerant to biologic therapy for UC (such as anti- TNF antibodies or anti-integrin antibodies) or to Janus kinase (JAK) inhibitors (such as tofacitinib). The medication used to qualify the patient for entry into this category must be approved for the treatment of UC. Investigators must be able to document an adequate clinical trial of the medication. Patients should fulfill 1 of the following criteria:   - Inadequate response: Signs and symptoms of persistently active disease despite induction treatment at the approved induction dosing that was indicated in the product label, or - Loss of response: Recurrence of signs and symptoms of active disease during approved maintenance dosing following prior clinical benefit (discontinuation despite clinical benefit does not qualify as having failed or being intolerant to UC biologic therapy), or - Intolerance: History of intolerance to infliximab, adalimumab, golimumab, vedolizumab, tofacitinib or other approved biologics or JAK inhibitors (including but not limited to infusion-related event, demyelination, congestive heart failure, or any other drug-related AE that led to a reduction in dose or discontinuation of the medication)   Patients previously exposed to biologic therapy who do not meet Inclusion Criterion 8b must still meet Inclusion Criterion 8a in order to be eligible to participate in the study.  Patients previously exposed to investigational therapies for the treatment of UC must still meet Inclusion Criteria 8a OR 8b  Patients who meet both Inclusion Criteria 8a and 8b will be considered to be “biologic-failed”, for the purpose of this study  **UC Medication Dose Stabilization Criteria**  9. Stable doses of the following drugs are permitted:  9a.  oral 5-ASA therapy: if the prescribed dose has been stable for at least 2 weeks prior to the screening endoscopy.  9b. oral corticosteroid therapy (prednisone ≤20 mg/day or equivalent, or budesonide extended release tablets 9 mg/day [budesonide MMX]); if the prescribed dose has been stable for at least 2 weeks before the screening endoscopy  9c. AZA, 6-MP, and methotrexate: if these immunomodulators have been prescribed at a stable dose for at least 8 weeks before the screening endoscopy  **Study Procedure Inclusion Criteria**  10. Are willing and able to complete the scheduled study assessments, including endoscopy and daily diary entry  11. Have clinically acceptable central laboratory test results at screening (retesting is allowed for hematology and chemistry), including:  11a. hematology: absolute neutrophil count ≥1.5 times (x) 109/L (≥1.5x103/μL or ≥1.5 GI/L), platelet count ≥100x109/L (≥100x103/μL or ≥100 GI/L), hemoglobin ≥8.5 g/dL (≥85 g/L) for males and >8.0 g/dL (>80 g/L) for females, lymphocyte count ≥500 cells/μL (>0.50x103/ μL or >0.50 GI/L), and total white blood cell count ≥3.0x109/L (≥3.0x103/μL or ≥3.0 GI/L)  11b. chemistry:   - serum creatinine ≤2x upper limit of normal (ULN) - total bilirubin level (TBL) ≤1.5xULN - alanine aminotransferase (ALT) and aspartate aminotransferase (AST) ≤2xULN - alkaline phosphatase (ALP) ≤1.5xULN - patients with an established diagnosis of Gilbert’s syndrome (requires source documentation showing unconjugated hyperbilirubinemia, with no evidence of hemolysis) can be included with bilirubin levels ≤3xULN | **Gastrointestinal Exclusion Criteria**  1.  Have a current diagnosis of Crohn’s disease, inflammatory bowel disease- unclassified (IBD-U) (formerly known as indeterminate colitis), or UC proctitis (disease limited to the rectum, that is, distal to the recto-sigmoid junction, which lies approximately 10-15 cm from anal margin)  2. Have an inherited immunodeficiency syndrome or a known monogenic cause of UC-like colonic inflammation  3. Previous bowel resection or intestinal or intra-abdominal surgery:   - have had extensive colonic surgery for UC or for other reasons (for example, subtotal colectomy), or are likely to require surgery for the treatment of UC during the study. Patients who have had limited colonic surgery (for example, segmental colonic resection) may be allowed in the study, if this does not affect the assessment of efficacy. Discussion with the sponsor should occur prior to screening of such patients - have had any small bowel or colonic surgery within 6 months prior to baseline - have had any non-intestinal intra-abdominal surgery within 3 months of baseline   4. Have evidence of toxic megacolon, intra-abdominal abscess, or stricture/stenosis within the small bowel or colon  **Adenoma, Dysplasia, and Gastrointestinal Cancer Exclusion Criteria**  5. Any history or current evidence of cancer of the gastrointestinal tract  6. Any current sporadic adenoma without dysplasia (adenomatous polyps occurring proximal to known areas of colitis) that has not been removed. Once completely removed, the patient is eligible for study  7. Dysplasia occurring in flat mucosa, sporadic adenomas containing dysplasia, and dysplasia-associated lesions or masses (DALMs) will be managed as follows:  7a. any history or current evidence of high-grade dysplasia is exclusionary  7b. any history or current evidence of dysplasia occurring in flat mucosa is exclusionary. This includes histopathology reporting “indefinite for dysplasia,” low-grade dysplasia, and high-grade dysplasia  7c. any history or current evidence of a nonadenoma-like DALM, with or without evidence of dysplasia, is exclusionary  7d. any current sporadic adenoma containing dysplasia or any current adenoma-like DALM that has not been removed is exclusionary. Once completely removed the patient is eligible for the study  **Criteria for Prohibited Medications**  8. Have received any of the following for treatment of UC within the time frames specified below:  8a. corticosteroid enemas, corticosteroid suppositories, oral budesonide standard formulation, or a course of IV corticosteroids within 2 weeks prior to screening endoscopy  8b. 5-ASA enemas or 5-ASA suppositories within 2 weeks prior to screening endoscopy  8c. immunomodulatory medications, including oral cyclosporine, IV cyclosporine, tacrolimus, mycophenolate mofetil, thalidomide or Janus kinase inhibitors (for example, tofacitinib) within 4 weeks prior to the screening endoscopy   - AZA, 6-MP, and methotrexate are allowed (see Inclusion Criterion 9b - other immunomodulatory medications should be discussed with the sponsor prior to screening   8d. anti-TNF antibodies (for example, infliximab, adalimumab, or golimumab) within 8 weeks prior to screening endoscopy  8e. anti-integrin antibodies (for example, vedolizumab) within 8 weeks prior to screening endoscopy  8f. agents that deplete B or T cells (for example, rituximab, alemtuzumab, or visilizumab) within 12 months of baseline. Patients remain excluded if there is evidence of persistent targeted lymphocyte depletion at the time of screening endoscopy  8g. any investigational nonbiologic therapy within 4 weeks prior to the screening endoscopy or within 5 half-lives prior to the screening endoscopy, whichever is longer  8h. any investigational biologic therapy within 8 weeks prior to the screening endoscopy or within 5 half-lives prior to the screening endoscopy, whichever is longer  8i. leukocyte apheresis (leukapheresis, for example, Adacolumn) within 3 weeks prior to screening endoscopy  8j. interferon therapy within 8 weeks prior to screening endoscopy  9. Have ever received anti-IL12p40 antibodies (for example, ustekinumab [Stelara®]) or anti-IL-23p19 antibodies (for example, risankizumab [BI-655066], brazikumab [MEDI-2070], guselkumab [CNTO1959], or tildrakizumab [MK-3222]) for any indication, including investigational use  10. Have failed 3 or more biologic therapies for UC  **Infectious Disease Exclusion Criteria**  11. Patients who:  11a. have evidence of active tuberculosis (TB), or  11b. have a past history of active TB, regardless of treatment, or  11c. are diagnosed with latent tuberculosis infection (LTBI) at screening  Patients diagnosed with LTBI at screening may be allowed to re-screen for the study, provided they fulfil the criteria described in Section 9.4.5.3. Patients who have a documented history of completing an appropriate TB prophylaxis regimen with no history of risk of re-exposure since their treatments were completed and no evidence of active TB are eligible to participate in the study  12. Have received a Bacillus Calmette-Guerin (BCG) vaccination within 12 months or received live attenuated vaccine(s) within 3 months of baseline or intend to receive such during the study  13. Have human immunodeficiency virus/acquired immune deficiency syndrome (HIV/AIDS) or test positive for HIV antibodies at screening  14. Have acute or chronic hepatitis B infection; or test positive for hepatitis B virus (HBV) at screening, which is defined as:  14a. positive for hepatitis B surface antigen (HBsAg+)  **OR**  14b. Negative for hepatitis B surface antigen (HBsAg-) and positive for anti-hepatitis B core antibody (anti-HBc+) in conjunction with detectable HBV DNA (see Section 9.4.5.4)  **OR**  14c. detectable HBV DNA (see Section 9.4.5.4)  15. Have current hepatitis C infection; or test positive for hepatitis C virus (HCV) at screening, defined as:   - positive for hepatitis C antibody and detectable HCV RNA (see Section 9.4.5.5)   16. Had Clostridium difficile or other intestinal infection within 30 days of screening endoscopy or test positive at screening for C. difficile or for other intestinal pathogens. Patients with a confirmed diagnosis of cytomegalovirus-associated colitis should have adequate treatment and resolution of symptoms at least 3 months prior to screening endoscopy  17. Patients with serious, opportunistic or chronic/recurring extraintestinal infections should be adequately treated and off antibiotics for 30 days without recurrence of symptoms prior to screening, including but not limited to the following:  17a. infections requiring IV antibiotics  17b. infections requiring hospitalization.  17c. infections that are considered “opportunistic” (examples are listed in Appendix 8)  17d. chronic, recurrent infections (eg. osteomyelitis, recurring cellulitis)  Patients with an opportunistic infection or chronic, recurrent infection (within the last 60 days) should be discussed on a case-by-case basis with the medical monitor  18. Patients with nonserious extraintestinal infections not adequately treated prior to screening  19. Have evidence of active/infectious herpes zoster infection ≤8 weeks prior to screening. Herpes zoster infections remain active until all vesicles are crusted over  **General Exclusion Criteria**  20. Have had lymphoma, leukemia, or any malignancy within the past 10 years. Exceptions: the following conditions are not exclusionary: a) basal cell or squamous epithelial carcinoma of the skin that has been adequately treated with no evidence of metastatic disease for 1 year, or b) cervical carcinoma in situ that has been adequately treated with no evidence of recurrence within the 3 years prior to baseline  21. Are investigator site personnel directly affiliated with this study and/or their immediate families. Immediate family is defined as a spouse, parent, child, or sibling, whether biological or legally adopted  22. Are Lilly employees or employees of third-party organizations involved with the study  23. Are currently enrolled in any other clinical study involving an investigational product or any other type of medical research judged not to be scientifically or medically compatible with this study  24. Have previously completed or discontinued from this study or any other study investigating mirikizumab. This criterion does not apply to patients undergoing rescreening procedures  25. Have had extra-abdominal surgery and have not recovered fully following surgery, including complete wound healing, before screening  26. Presence of significant uncontrolled neuropsychiatric disorder or judged at risk of suicide in the opinion of the investigator;  **OR**  marked “yes” to Columbia-Suicide Severity Rating Scale (C-SSRS) Question 4 or 5 on ideation during the screening period prior to dosing at Visit 1;  **OR**  marked yes to C-SSRS suicide behaviors questions during the screening period prior to dosing at Visit 1;  **AND**  the ideation or behavior occurred within the past month  27. Have an unstable or uncontrolled illness, including but not limited to cerebrocardiovascular, respiratory, gastrointestinal (excluding UC), hepatic, renal, endocrine, hematologic, or neurological disorders that would potentially affect patient safety within the study or confound efficacy assessment. Patients requiring systemic corticosteroids for non-UC conditions (except corticosteroids to treat adrenal insufficiency) are excluded  28. Have a known hypersensitivity to any component of this investigational product  29. Have a solid organ transplant or hematopoietic stem cell transplantation  30. Are unwilling or unable to comply with the use of a data collection device to directly record data from the patient daily for the duration of Study AMAN, or unable to complete other study procedures  31. Are unsuitable for inclusion in the study in the opinion of the investigator or sponsor for any reason that may compromise the subject’s safety or confound data interpretation  32. Unable to complete study procedures  33. Are pregnant, breastfeeding, or planning pregnancy (women only) while enrolled in the study, or within 20 weeks after receiving the last dose of study agent  34. Current or history of alcohol dependence and/or drug abuse within the last year |
